# Amino Acids in the Development of Prodrugs

**DOI:** 10.3390/molecules23092318

**Published:** 2018-09-11

**Authors:** Nuno Vale, Abigail Ferreira, Joana Matos, Paula Fresco, Maria João Gouveia

**Affiliations:** 1 Laboratory of Pharmacology, Department of Drug Sciences, Faculty of Pharmacy, University of Porto, Rua de Jorge Viterbo Ferreira 228, 4050-313 Porto, Portugal; abigail.ferreira@hotmail.com (A.F.); pfresco@ff.up.pt (P.F.); 2 Institute of Molecular Pathology and Immunology of the University of Porto (IPATIMUP), Rua Júlio Amaral de Carvalho, 45, 4200-135 Porto, Portugal; 3 Instituto de Investigação e Inovação em Saúde (i3S), University of Porto, Rua Alfredo Allen, 208, 4200-135 Porto, Portugal; mariajoaogouveia@gmail.com; 4 Department of Molecular Pathology and Immunology, Institute of Biomedical Sciences Abel Salazar (ICBAS), University of Porto, Rua de Jorge Viterbo Ferreira 228, 4050-313 Porto, Portugal; 5 LAQV&REQUIMTE, Laboratory of Applied Chemistry, Department of Chemical Sciences, Faculty of Pharmacy, University of Porto, Rua de Jorge Viterbo Ferreira 228, 4050-313 Porto, Portugal; 6 SpiroChem AG, Rosental Area, WRO-1074-3, Mattenstrasse 24, 4058 Basel, Switzerland; joana.c.m.matos@gmail.com

**Keywords:** amino acid transport, drug delivery, prodrug design, stability studies, in vitro and in vivo assays

## Abstract

Although drugs currently used for the various types of diseases (e.g., antiparasitic, antiviral, antibacterial, etc.) are effective, they present several undesirable pharmacological and pharmaceutical properties. Most of the drugs have low bioavailability, lack of sensitivity, and do not target only the damaged cells, thus also affecting normal cells. Moreover, there is the risk of developing resistance against drugs upon chronic treatment. Consequently, their potential clinical applications might be limited and therefore, it is mandatory to find strategies that improve those properties of therapeutic agents. The development of prodrugs using amino acids as moieties has resulted in improvements in several properties, namely increased bioavailability, decreased toxicity of the parent drug, accurate delivery to target tissues or organs, and prevention of fast metabolism. Herein, we provide an overview of models currently in use of prodrug design with amino acids. Furthermore, we review the challenges related to the permeability of poorly absorbed drugs and transport and deliver on target organs.

## 1. The Prodrug Concept: An Overview

**Figure 1:**
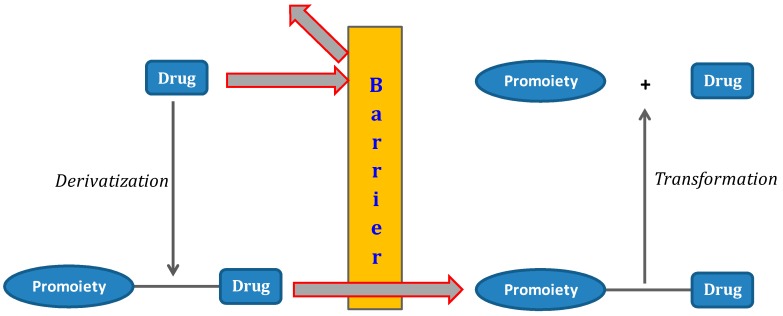
Illustration of prodrug concept. (Adapted from Ref. [5]).

The term prodrug was introduced in the 1950s to describe a covalent link between a drug and a chemical moiety. The term is also used to characterize some salt forms of the active drug molecule [1]. Many drugs see medicinal use denied due to poor physical, pharmacokinetic or pharmacodynamic properties. Therefore, the use of different strategies to improve solubility, stability, permeability and targeting problems in drug discovery and development is crucial. Since prodrugs might alter the tissue distribution, efficacy and toxicity of the parent drug, the implementation of a prodrug approach in the early stages of drug development is a growing trend [2,3,4,5]. Prodrugs are bio-reversible derivatives of drug molecules that undergo enzymatic and/or chemical transformation to release the parent drug [3]. There are several pro-moieties that can be used in prodrug design, and its selection is a crucial step. The goal is achieving a chemically stable prodrug that after bioconversion does not generate toxic metabolites [2]. Ideally, the prodrug should be converted into the parent drug as soon as the goal is achieved, followed by rapid elimination of the released derivatizing group (Figure 1).

## 2. Amino Acids and Routes of Transport

Drug-delivery is a cascade of molecular migration processes, in which the active principle is exposed to several biological media with various hydrophilic and lipophilic characters. Membrane penetration is controlled by several physicochemical parameters, with the most promising ones being species-specific basicity and lipophilicity. The latter property has a great importance in several scientific areas such as pharmacy, bio- and medicinal chemistry since it expresses the affinity of the molecule for a lipophilic environment [6].

One of factors that determine drug bioavailability is related to transport across biological membranes, therefore drug permeability is a crucial parameter for its effectiveness and is directly related to their physicochemical characteristics [7,8]. Water solubility and lipophilicity strongly influence drug interactions with components of the absorptive surface within the gastrointestinal tract, giving rise to differential preferences in terms of transport pathways. In many cases, drugs of poor aqueous solubility exhibit a high degree of lipophilicity, which could be demonstrated by solubilization of lipophilic compounds with monoglycerides and free fatty acids, and liposomes [9,10].

One strategy for increasing water-solubility is the use of amino acids as moieties. Amino acids are basic constituents of a cell structure but require specialized transport systems to cross the plasma membrane. Several of these transport systems have been identified and classified based on their substrate affinity, dependence on sodium ions, energy and pH [11]. Amino acids are pharmacological agents with low toxicity which makes them attractive carriers for development of prodrugs of poorly absorbed therapeutic agents. In addition, an amino acid promoiety improves the water solubility of the prodrug [12]. The importance of these transporters in pharmacokinetics has been recognized through several studies that report an improved bioavailability of amino acid linked compounds [11].

L-type amino acid transporters 1 (LAT1) and 2 (LAT2) are responsible for carrying large neutral amino acids from extracellular fluids into the cells, and hence their involvement in pharmacokinetics [13]. LAT1 is a transmembrane protein abundantly expressed at the blood-brain barrier (BBB), where it ensures the transport of hydrophobic acids from the blood to the brain. The natural substrates of LAT1 are large neutral amino acids such as *L*-leucine, *L*-tryptophan, and *L*-phenylalanine. These compounds have excellent affinity for the LAT1 transporter protein and thus rapidly pass through the BBB. Therefore, LAT1 is an interesting target for drug delivery to the central nervous system (CNS) [14]. Also, solubility and permeability interactions and their impact on intestinal drug absorption are most prominently described by the Biopharmaceutics Classification System (BCS) [15].

Previously, the transport systems for peptides, amino acids and nucleoside/nucleobase on the corneal epithelium was reported [16]. These transport mechanisms can be used for targeting drug delivery [2,17,18]. Amino acid prodrug strategies utilizing endogenous nutrient transporters that are over expressed in certain organs have become an attractive approach for improved and targeted drug delivery [4]. Among nutrient transporters, those transporting amino acids are preferred for drug delivery due to their ubiquitous nature and overlapping substrate specificity [17,18]. They are known to transport not only naturally occurring amino acids but also amino acid related compounds, making them suitable delivery targets for amino acid-based drugs and prodrugs [17]. Moreover, amino acids are building blocks for proteins and are generally regarded as safe [4,19].

The intention of this work is to review and summarize the use of amino acids in drug design in order to improve drug solubility and delivery.

## 3. Drugs and Diseases

### 3.1. Anti-Viral Drugs

#### 3.1.1. Herpes Viruses Infections (*Herpes Simplex* or *Zoster* and *Cytomegalovirus*)

**Figure 2:**
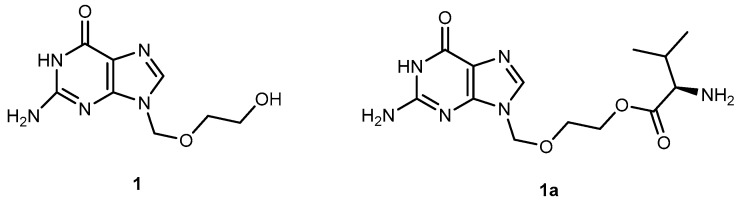
Chemical structures of ACV (**1**) and the amino acid prodrug valacyclovir (**1a**).

The anti-viral drug acyclovir (ACV **1**, Figure 2) has poor oral bioavailability due to the lack of a transport system on the intestinal tract capable of recognizing this drug as a substrate [20]. The attempt to improve this characteristic resulted in the preparation of several prodrugs of **1**. Different approaches have been implemented to achieve this goal [21]. Colla et al. and Maudgal et al. used amino acid moieties to prepare prodrugs of **1** which were potentially useful in the preparation of eye drop formulations [22,23]. A similar approach was used by Beauchamp et al. in order to improve oral administration. With a different purpose, Shao et al. and Yang et al. reported the synthesis, nasal and ocular absorption, and metabolism of aliphatic acid ester prodrugs of **1** [24]. Recently, Gao and Mitra have focused their work on the improvement of membrane transport of prodrugs of **1**. Their studies resulted in the preparation of a series of N-acyl-ACV, α, β and γ-amino acid esters and dicarboxylic acid esters of **1** [21,25].

**Figure 3:**
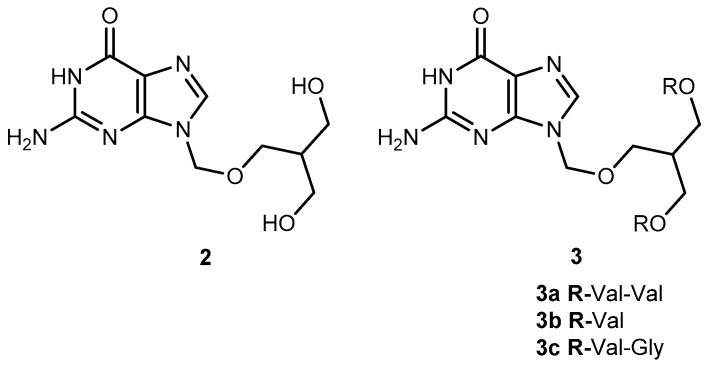
Ganciclovir and its diester prodrugs.

Valacyclovir (**1a**, Figure 2), an *L*-valyl ester of **1** is rapidly converted to the parent drug after oral administration, increasing the bioavailability of the parent drug by 3- to 5-fold [20,26,27,28]. This improvement results from the transport of **1a** by the peptide transport PEPT1, abundantly expressed in the intestine [20,29,30,31,32,33]. It is important to notice that a peptide bond is not required for recognition of a prodrug by the peptide transporter [31]. The study performed by Katragadda and co-workers with amino acid derivatives of **1** demonstrated an upgrade in the ocular bioavailability of **1** of approximately 2-fold after topical administration of *L*-serine and *L*-valyl esters of **1** [17]. Drug delivery to different eye structures (retina, choroid, vitreous humor and sclera) is a challenging task for the pharmaceutical industry. Another example is the drug ganciclovir (GCV; **2**, Figure 3) used in the treatment of cytomegalovirus (CMV) retinitis, that presents poor aqueous solubility [34]. Consequently, in these infections, frequent administration of the drug or surgery are necessary, which turns so crucial the development of strategies to enhance permeation of **2** into corneal or retinal tissues.

Peptide transporters (PEPT1 and PEPT2) are widely used as targets in drug design. In fact, *L*-valine ester prodrugs of **2** and **1** presented an intestinal absorbance enhancement as a result of PEPT1 mediated transport [34]. This peptide transporter is expressed in the corneal epithelium, and dipeptide derivatives of **1** have shown a better permeation in this tissue [29,34]. The physicochemical properties of di-ester prodrugs of **2** (Figure 3) were studied by Patel’s research group, and most of the tested compounds showed good affinity to PEPT1. Moreover, solubility and lipophilicity properties make the derivatives **3a**, **3b** and **3c** good candidates for drug delivery [34]. The peptide approach allowed an improvement in the prodrugs lipophilicity without compromising their solubility. In fact, **3b** showed a 42 times higher solubility compared to the parent drug. It is also interesting that the di-ester prodrugs did not show a greater selectivity to the virus but were found to be more potent. This fact may be explained by the higher binding affinity of these derivatives for the peptide transporters [34].

**Figure 4:**
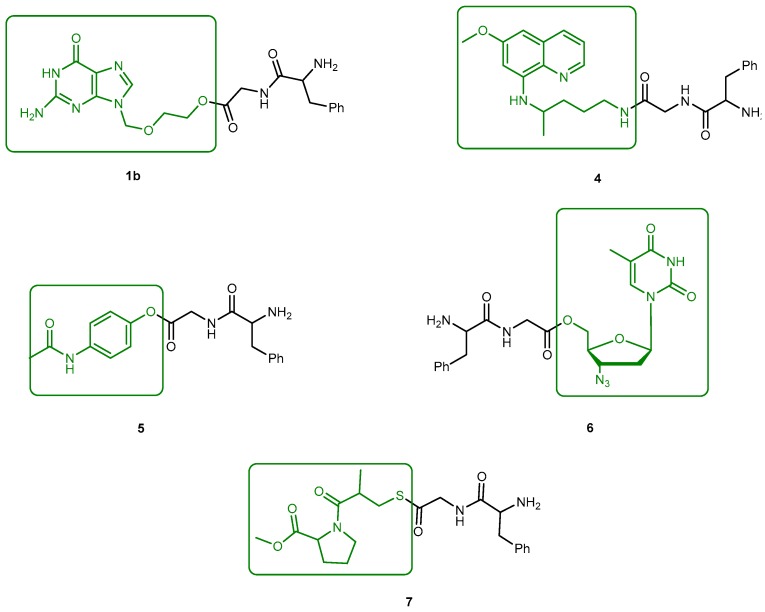
The Phe-Gly derivatives of **1a**, primaquine (**4**), paracetamol (**5**), azidothymidine (**6**) and captopril methyl ester (**7**). The parent drugs are highlighted in green.

The problems associated with antiviral drugs are of great concern. The large number of herpetic virus widespread in nature is responsible for many viral diseases affecting humans. Progress has been made on the improvement of **1**-based therapies. From a clinical point of view, this research has, so far, culminated in the *L*-valyl ester prodrug **1a**. At present, there are few studies related to dipeptide derivatives of **1**. Santos et al. evaluated the chemical stability, solubility, and antiviral activity of dipeptide derivatives of **1**. The principal use of the dipeptide-based prodrug approach occurs in drugs with an alcohol functional group [35]. However, it is important to understand how this approach works for other drugs with different functional groups. Santos et al. also studied the effect of functional groups, as the thiol and amine groups, on the chemical and enzymatic reactivity of the resulting prodrug [35]. The Phe-Gly dipeptide derivatives of **1**, **1b**, primaquine (**4**), paracetamol (**5**) and azidothymidine (**6**) and captopril methyl ester (**7**) were evaluated (Figure 4).

**Scheme 1:**
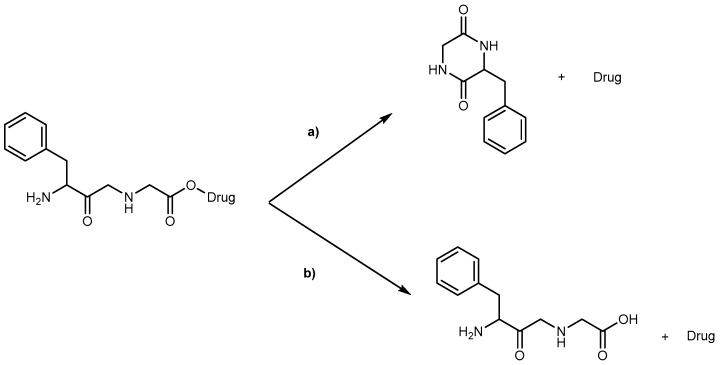
General degradation pathways of dipeptide prodrugs in human plasma [35].

Kinetics studies at pH 7.4, showed that the derivative **1b** is degraded via two parallel pathways (Scheme 1). The major degradation pathway, (a), results in the formation of a diketopiperazine, which is consistent with the intramolecular cyclization expected for dipeptide alkyl and aryl esters [36].

However, high-performance liquid chromatography (HPLC) analysis showed that the dipeptide may also degrade through direct ester hydrolysis-pathway (b). Moreover, it has been established that the drug’s leaving group influences the ratio between the two different pathways. In fact, degradation of derivatives **5** and **7** resulted in the quantitative formation of the diketopiperazine and parent drug. On the other hand, derivative **6** indicates that direct hydrolysis was also operating on the drug release pathway, suggesting that less bulky alcohols are more susceptible to ester hydrolysis [35,37]. When studying their degradation in human plasma, all derivatives released the correspondent parent drug and the dipeptide (Phe-Gly) moiety with the exception of derivative **4** which degradation resulted in the formation of the correspondent amino acid amide (Gly-PQ) as a reaction intermediate. Both results are consistent with the enzymatic degradation of dipeptide prodrugs via stepwise removal of amino acid residues and direct cleavage of the dipeptide carrier. Derivatives degradation has been shown to be influenced by the nature of the drug’s leaving group and the rate at which the drug is released. This implies that this approach is chemical and enzymatically more adequate for drugs containing alcohol or amine functional groups [35].

In general, the synthesis of prodrugs to improve bioavailability is related to the affinity for peptide transporters. Although high affinity does not necessarily imply the transport of the drug, it is essential to bioavailability improvement. Therefore, both bioavailability and pharmacokinetic studies are needed to determine a lead prodrug. Accordingly, Thomsen et al. compared the bioavailability in rats and the transport in CaCo-2 cells of prodrugs of **1** and showed that the high affinity of Glu-ACV-Sar prodrug for PEPT1 in CaCo-2 cells was not synonymous of prodrug translocation by the peptide transporter [38]. On their experiments, intracellular accumulation of Glu-ACV-Sar was studied in comparison with **1** and **1a**, as a measurement of **1** in cell extracts, and they observed that neither the prodrug nor **1** was detected when applying Glu-ACV-SAR, indicating that Gly-ACV-Sar is not translocated by PEPT1 [38]. The high affinity of the prodrug for the peptide transporter did not result in a higher bioavailability of the parent drug. The opposite was even observed for the prodrug **1a**. These studies are very important in case of drugs used in immunocompromised cancer patients since the side effects of chemotherapy can influence drug absorption and so drugs/prodrugs with more reliable absorption are of particular value [39]. Different studies indicated that bioavailability of **1** is equal after administration of **1a** in immunocompromised cancer patients and in healthier volunteers [39,40].

Han et al. went further on the study of amino acid prodrugs. They studied the permeability and hydrolysis of amino acid ester prodrugs in CaCo-2/hPEPT1 cells, not only for derivatives of **1** but also to amino acid derivatives of azidothymidine [31,33]. Again, the *L*-valyl ester of azidothymidine and **1** were the derivatives with higher membrane permeabilities. An interesting fact is that the *L*-configuration of the amino acid is more suitable for the prodrug strategy. Comparing *L*- and *D*-valyl ester derivatives of **1**, the *L*-valyl presents the higher permeability and faster conversion relative to the parent drug [33,34].

**Figure 5:**
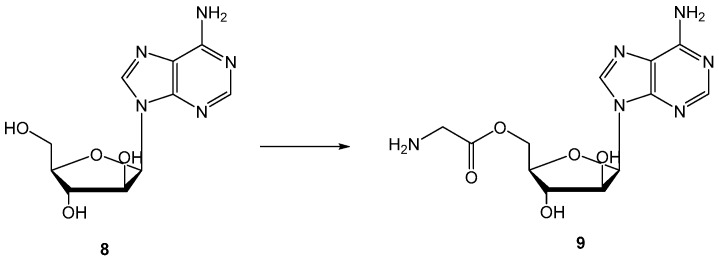
Chemical structures of **8** and a synthesized amino acid ester.

Another issue was approached by the Hilfinger research group: they used the prodrug strategy as a protection from metabolic conversion using vidarabine (**8**, Figure 5) as a drug and different *D*- and *L*-amino acid esters were synthesized and evaluated (Figure 5) [41]. Data obtained demonstrate that substitution of 5′-OH group results in the enhanced uptake of prodrugs and parent drug, and protection of the prodrug from deamination before hydrolysis to **8**.

**Figure 6:**
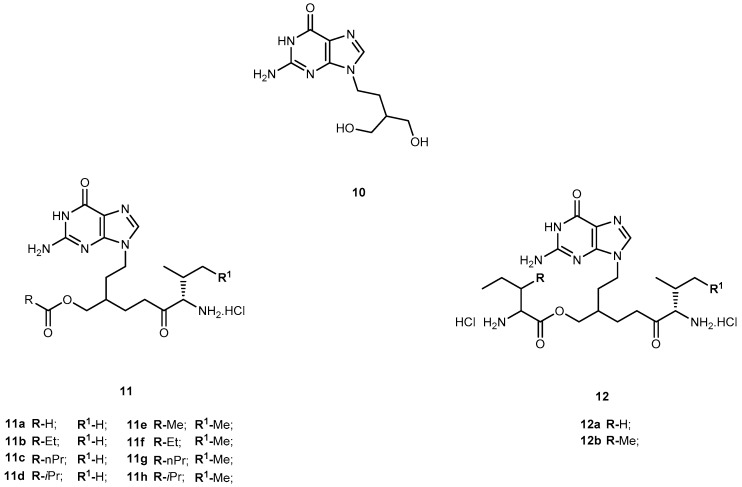
Structures of penciclovir (**10**) and its amino acid ester prodrugs **11** and **12**.

Penciclovir (9-(4-hydroxy-3-hydroxymethylbut-1-yl)guanine, **10**, Figure 6) is a potent and highly selective inhibitor of the replication of herpes virus, including herpes simplex virus type 1 and 2 (HSV-1 and HSV-2), varicella-zoster virus (VZV) and *Epstein-Barr* virus (EBV) both in cell cultures and in animals [42]. The main advantage of **10** over **1** is that its antiviral activity in cell culture is more persistent. However, like other acyclic nucleoside analogs (described above), **10** has poor oral bioavailability [43]. In order to overcome this, Kim et al. synthesized the *O*-acyl-*O*-amino acid esters as potential prodrugs of penciclovir. The two naturally occurring branched chain amino acyl groups, *L*-valyl and *L*-isoleucyl, were selected for the protection of one of the two hydroxyl groups. As previously shown they have an optimal combination of the side chain length and degree of branching for the oral bioavailability and chemical stability in the amino acid ester prodrug acyclovir [44,45]. All amino acid ester prodrugs **11a**–**d**, **12a**–**b** (Figure 6) were highly soluble in water, showing a remarkable increase in aqueous solubility, compared with the parent drug.

The in vitro antiviral activity of the prodrugs synthesized against HSV-1 in Vero cells were compared with thereof **10**. Compounds **11a**, **11f**, and **11d** showed no significant antiviral activity at a concentration of 100 μM. Although compounds **11c**, **11g**, **12a**, **12b** were active against HSV-1, their antiviral activity was approximately 3- to 5-fold lower than **10**. Kim et al. assessed the bioavailability of prodrugs of **10** after a single oral administration through determination of the total amount of **10** recovered in the urine over 48 h. Of the prodrugs tested, **11a** present a bioavailability approximately 4-fold higher comparatively to that of **10** [45]. The introduction of amino acid pro-moieties not only increase antiviral activity, but also improve prodrugs bioavailability, compared to the parent drug.

**Figure 7:**
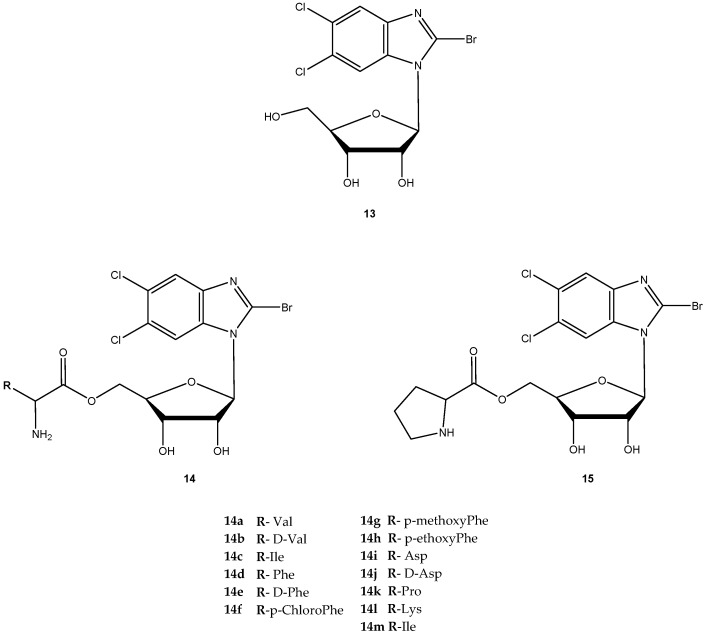
Structures of novel amino acid ester prodrug of **13**.

The antiviral agent 2-bromo-5,6-dichloro-1-(β-d-ribofuranosyl)benzimidazole (**13**, Figure 7) is a member of a novel class of benzimidazole ribonucleosides that are potent inhibitors of human cytomegalovirus (HCMV) replication, with lower cellular toxicity at concentrations inhibiting viral growth [46]. The antiviral mechanism of action of **13** is unique and involves inhibition of viral DNA processing virus assembly [47]. Like certain other nucleoside agents, **13** exhibits modest oral bioavailability due to extensive first pass effect, possibly related to its glycoside bond [48]. Song et al. synthesized amino acid ester prodrugs of **13** (**14a**–**l** and **15**) in order to (a) investigate their affinity for and transport by hPEPT1 and (b) determine if the promoiety affords stabilization of glycoside bond. For the synthesis of novel prodrugs, the amino acids were used as pro-moieties including aliphatic amino acids and its analogs, secondary amino acids and polar amino acids (Figure 7) [48].

The prodrugs of **13** were evaluated for their affinity for hPEPT1 by evaluation of their ability to inhibit [^3^H]glycylsarcosine ([^3^H]Gly-Sar) uptake in HeLa cells overexpressing hPEPT1 transporter. With exception for **14b**, **14k**, **14l** and **14j**, all prodrugs exhibited significantly higher affinity for hPEPT1 than the parent drug. Additionally, these authors also observed that *L*-amino acids exhibited 1.3- to 2-fold higher affinity for hPEPT1 than their counterparts [48]. It was hypothesized that amino acid ester prodrugs can enhance both in vitro potency and systemic exposure of **13** through evasion of its metabolizing enzymes. To test this hypothesis, the authors tested eight different amino acid prodrugs of **13** (**14a**, **14b**, **14c**, **14i**, **14j**, **14k**, **14l** and **14m**) for *N*-glycosidic bond stability, ester bond stability, Caco-2 cell uptake, antiviral activity in HCMV-infected cell system and cytotoxicity [49]. The prodrugs were resistant to metabolism by metabolizing enzymes, and ester bond cleavage was rate-limiting in the metabolic formation of the prodrug.

Although **13** was metabolized by the DNA repair enzymes hOOG1 and mMPG, their amino acid ester prodrugs were not a substrate of these enzymes, which suggest that prodrugs of **13** may evade metabolizing enzymes encountered in first pass organs and other tissues. Thus, metabolism of **13** could be controlled by the selection of the promoiety. The observation that high ester bond stability conferred greater *N*-glycosidic bond stability to prodrugs suggested that prodrug ester bond cleavage must take place before *N*-glycosidic bond cleavage occurs. From all prodrugs tested, **14i** was 3-fold more selective than the parent drug for inhibition of HCMV replication.

**Figure 8:**
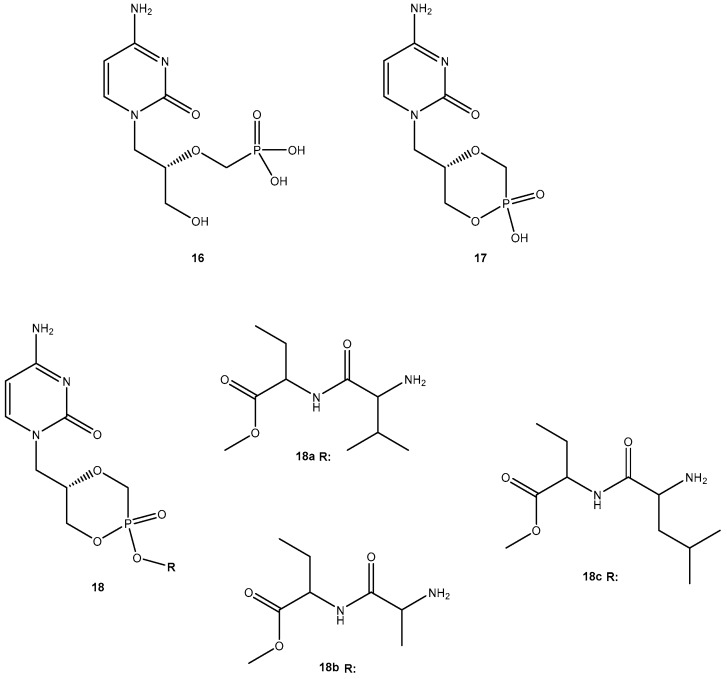
Structures of cidofovir (**16**), ciclocidofovir (**17**) and its dipeptide derivatives **18a**–**c**.

Due to its potency, selective antiviral activity, stability profile and greater half-life (5-fold than **13**), **14i** was considered an excellent candidate for in vivo assessment and pharmacokinetic comparison with the parent drug. The ability to evade of metabolizing enzymes in vitro of the amino acid ester prodrug of **13**, might provide a modular approach for translating this in vitro stability into enhanced in vivo delivery of **13** [49]. Unfortunately, until present, there are no in vivo studies that corroborate this hypothesis. Cidofovir (**16**, Figure 8) is a broad-spectrum antiviral agent used to treat Acquired Immunodeficiency Syndrome (AIDS)-relative CMV retinitis, and a promising drug active against variola and other orthopoxviruses. However, its low oral bioavailability led to the development of novel analogs in order to improve their transport properties [50]. MacKenna et al. used a prodrug strategy targeting the peptide transporter-1 (hPEPT1) using cyclic cidofovir (**17**, Figure 8), which is a prodrug of **16**. After conversion of **17** in cells by cyclic AMP phosphodiesterase, their products were used to prepare dipeptides derivatives. The results obtained for derivatives of **17** were compared with those previously obtained for dipeptide derivatives of **16**. The in vitro transport evaluation of the **18a**–**c** showed an enhancement in permeability compared to **16** and **17**. Therefore, the derivatives of the valine amino acid were the best of the tested prodrugs [50] suggesting that this amino acid improves affinity to hPEPT1.

#### 3.1.2. Human Immunodeficiency Virus (HIV)

Nucleoside analogs continue to play an important role as antiviral agents [51]. Nonetheless, the evaluation of several nucleoside analogues as antiretroviral chemotherapeutic agents has revealed potential drug delivery problems related to its rapid metabolism and low availability [52].

**Figure 9:**
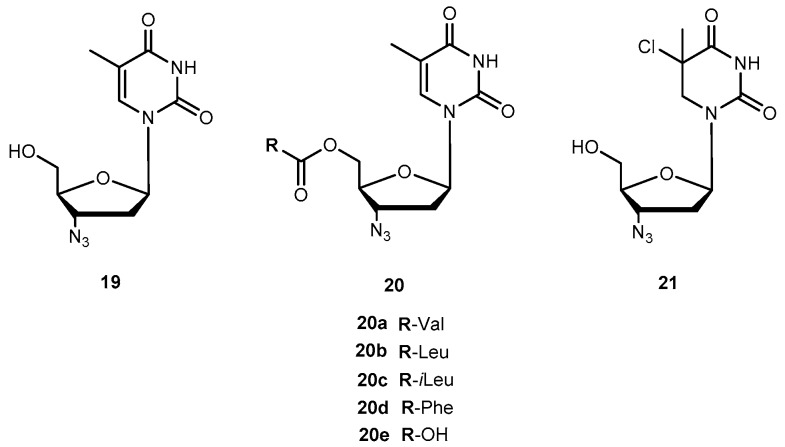
Chemical structures of **19** and its amino acid ester prodrug.

Zidovudine (AZT, 3′-azido-3′-deoxythymidide, **19**, Figure 9) was the first 2′-3′-dideoxy-nucleoside (ddN) approved by US Food and Drug Administration (FDA) for treatment of patients suffering from AIDS [53,54]. The mechanism of action of **19** involves its conversion into the corresponding 5′-*O*-triphosphate, which inhibits the replication of the virus by competitive inhibition of the viral reverse transcriptase (RT) and by incorporation and subsequent chain termination of the growing viral strand [55]. Administration of **19** is frequently associated with a significant dose-dependent toxicity, additionally to its short half-time in human plasma which requires frequent administration to maintain therapeutic drug concentrations [56,57]. Efforts have continuously been made to improve some therapeutic characteristics of **19**, most of them focused on prodrug design. Turk et al. synthesized six novel amino acid ester prodrugs of **19** (**20a**–**f** and **21**, Figure 9) using 5′-*O*-ester substitution strategies and evaluated their anti-HIV activity and cytotoxicity on different cell cultures [58].

**Figure 10:**
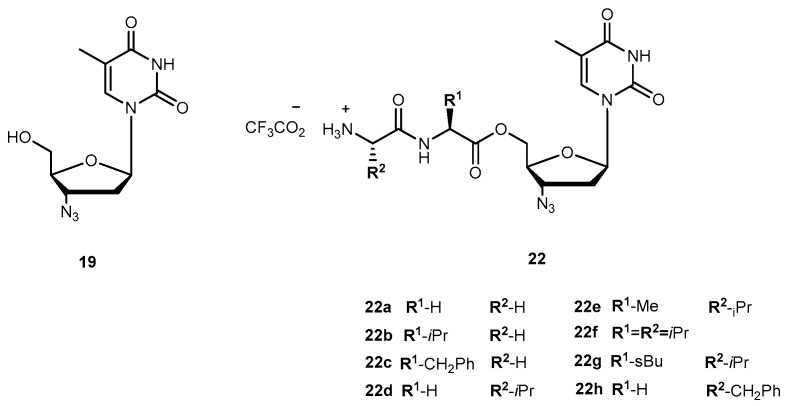
Structure of **19** and its dipeptide prodrugs **22a**–**h.**

Cytotoxicity and inhibition assays were performed in two different cell types: peripheral blood mononuclear cells (PBMCs) and MT2. During evaluation of cell viability, the compounds also showed a dose-dependent cytotoxicity. Although parameters obtained when using different methodologies cannot be directly compared, their correlation was significant. In one or both cell cultures used, all the prodrugs synthesized have a similar or higher selectivity index than **19** itself, with the exception of **20f** which showed no anti-HIV activity and **20d** that showed a diminished capacity to inhibit HIV replication. These facts could be directly related to the high acidity of their amino acids or higher volume. However, neither acidity level nor volume explains the activity variation showed by the rest of the compounds. Authors hypothesized that it could be due to different transport mechanisms depending either on the amino acids involved (for special transport) or lipophilicity of the substituent that might be implicated in each case. **20a** and **20c** demonstrated an improved ability to inhibit HIV replication on both cell types studied. Nonetheless, using amino acids to design **19** derivatives result in increased activity of parent drug [58]. Santos et al. also designed new analogues of **19** using 5′-*O*-ester substitution, however, instead of single amino acid, they used dipeptides (**22a**–**h**, Figure 10) to target the human intestinal oligopeptide transporter hPEPT1 and evaluated their stability at pH 7.4 in buffer and human plasma, cytotoxicity and retroviral activity [59].

**Scheme 2:**
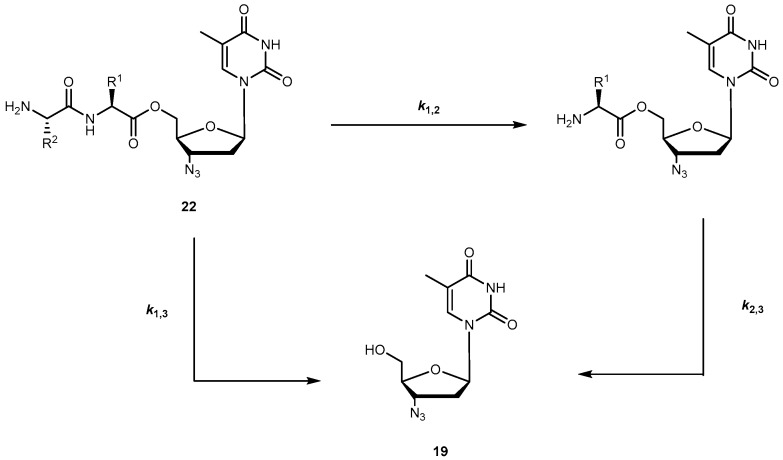
Pathways observed for releasing of **19** by dipeptide esters **22** in human plasma (reproduced from [59]).

The dipeptide esters of **19** undergo cyclization in buffer (pH 7.4) to release the parent drug at a rate that depends on the size of the chains of the peptide carrier. Santos et al. observed that the prodrugs were considerably more stable if the bulky α-branched amino acids (e.g., Ile and Val), were present, particularly, as C-terminal residues. In fact, substrate half-lives in plasma were remarkably higher in the presence of hydrophobic α-branched amino acids. In fact, esters were also good substrates for hPEPT1 in vitro, with Val-Gly-AZT and Val-Ala-AZT presenting the highest affinity to the transporter. They also showed that incubation in human plasma revealed that most of the dipeptide esters of **19** release the parent drug through two aminopeptidases-mediated pathways: (1) stepwise cleavage of each amino acid and (2) direct cleavage of the dipeptide drug ester bond (Scheme 2) [59].

The selectivity index of the prodrugs was 2- to 3-fold higher than that of **19** for all compounds analyzed, which indicates that these are potential dipeptide-based carriers for development of effective antiviral drug-delivery system [59].

**Figure 11:**
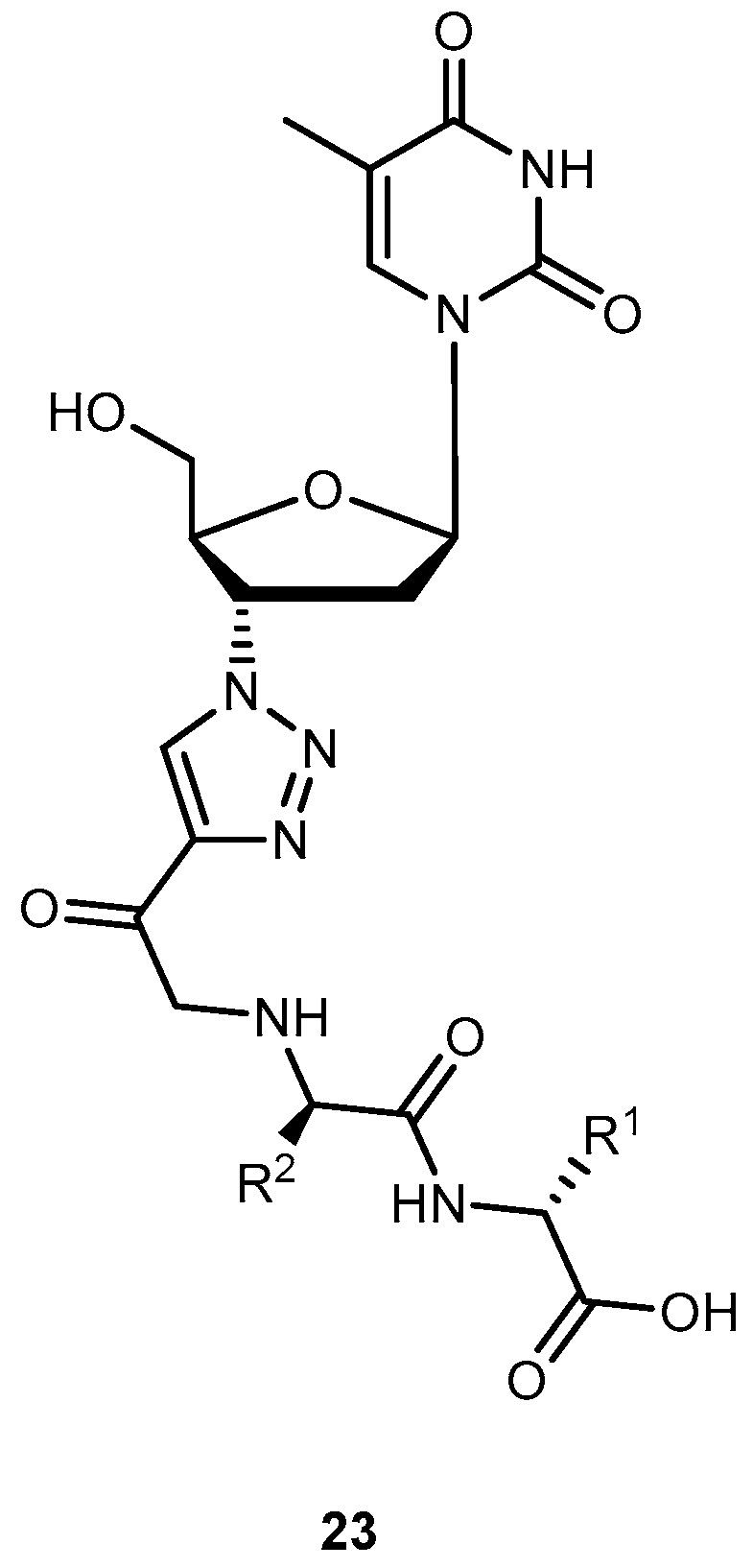
The general structure of dipeptide conjugates of **19** designed by Zhang et al. [60].

Using a similar design strategy, Zhang et al. developed a focused dipeptide conjugated of **19** libraries and screened in a PEPT1 overexpressing cell model in order to assess their abilities to compete with the known ligand cephalexin [60]. The 61 dipeptide conjugates of **19** synthesized consisted of different combination of amino acids. Their general structure is depicted in Figure 11.

Not all the 20 natural amino acids were included. The amino acids Asn, Gln, Asp, Met, Cys, His, Trp and Tyr were excluded because they are not very stable in physiological environment. However, authors tried to include most of the amino acid that had been reported to be involved in the PEPT1 activity namely Phe, Val, Leu, Ile, Gly and Ala. To evaluate the bioactivities of the entire library as PEPT1 substrates, authors used adenovirus Ad.RSVhPepT1 transfected HeLa cells that transiently overexpressed PEPT1. After evaluating their cytotoxicity, compounds yielding a cell viability greater than 85% were included in the cephalexin competition study. Several dipeptide sequences as Ser-Glu and Pro-Ile were found to have high affinity to PEPT1 and to mediate significant active transport activity across intestinal epithelia [60].

**Figure 12:**
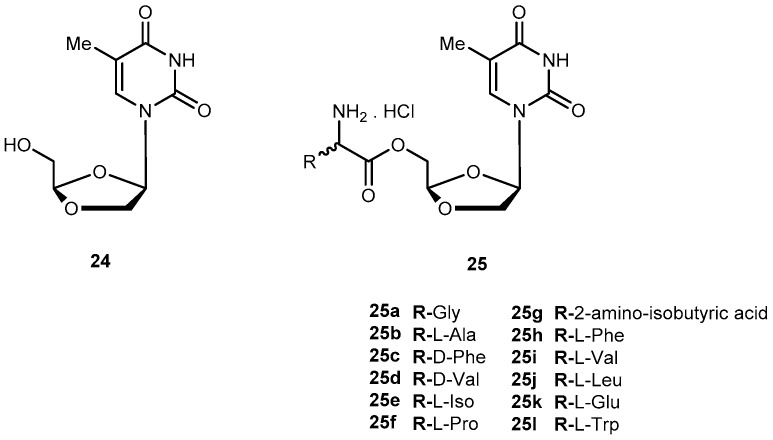
Structures of 5′-*O*-aminoacid esters of DOT.

DOT ((−)-β-d-(2R,4R)-dioxolane-thymine, **24**, Figure 12) is a pyrimidine 1,3-dioxolane nucleoside, exhibiting interesting activity against drug-resistant mutants of HIV-1. The use of amino acids for a prodrug strategy also provides advantages in the cellular transport system [61]. In view of this information, Liang et al. synthesized 5′-*O*-amino acid ester prodrugs of **24** (**25a**–**l**, Figure 12) and evaluated their anti-HIV activity in vitro. Most of the synthesized prodrugs exhibited potent anti-HIV activity against HIV-1_LAI_ in PBMC cells, without increased cytotoxicity in comparison to the parent drug.

Despite prodrugs exhibited good chemical stability with half-lives from 3 h to 54 h at phosphate buffer (pH 2.0 and 7.4), they were labile to porcine esterases with lower half-lives (12.3 to 48 min). The results suggest that prodrugs are effective substrate for porcine esterase. Overall, some class of prodrugs of **24** may improve the overall biological properties of the parent drug [62].

**Figure 13:**
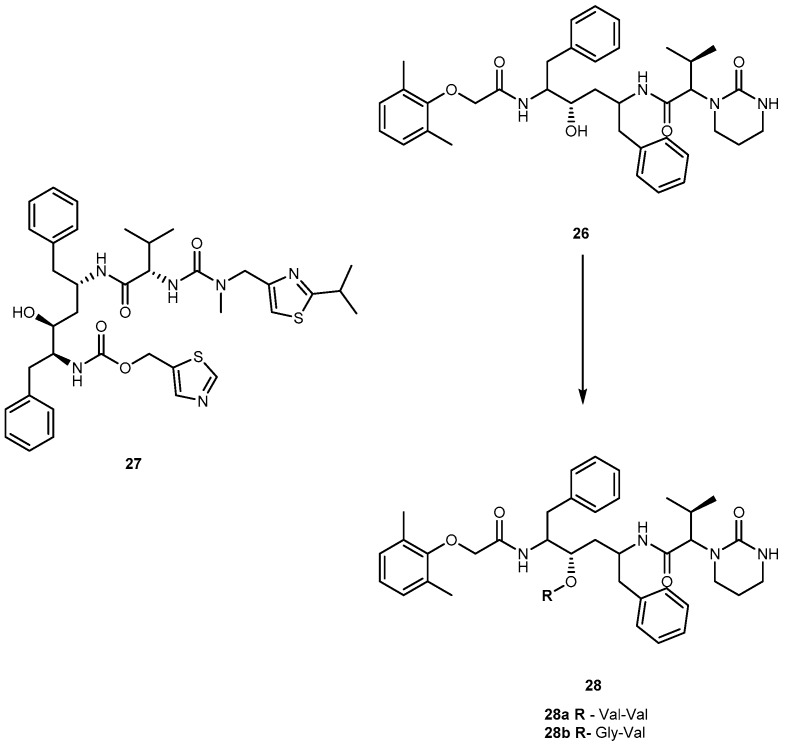
Structures ritonavir (**27**) lopinavir (**26**) and its dipeptides prodrugs (**28a** and **b**).

Lopinavir (**26**, Figure 13), an analog of ritonavir (**27**, Figure 13), is a protease inhibitor (PI) indicated for the treatment of HIV infection. When administrated alone, **26** exhibits low oral bioavailability in rats and humans and, usually, it is taken in combination with **27** (Kaletra^®^). Most likely, its low bioavailability may be related to extensive liver metabolism by CYP3A4 [63]. The compound **26** is prevented from entering the cells by membrane efflux pumps, such as P-glycoprotein (P-gp) and multidrug resistant protein (MRP2). This process diminishes its plasma concentration and thereby decreases its anti-HIV efficacy. In an attempt to prevent the first-pass metabolism and efflux of **26**, Argawal and co-workers synthesized dipeptide prodrugs of **26**, Val-Val-LVR **28a** and Gly-Val-LVR **28b** (Figure 13).

Solubility studies indicate that these prodrugs increase aqueous solubilities in comparison to parent drug that is almost insoluble in water. Moreover, prodrugs did not exhibit significant cytotoxicity to cells at concentrations ranging 5–50 μM. Although at higher concentrations (100 and 200 μM) they were significantly cytotoxic to the cells. Accumulation and transport data of **28a** and **28b** across MDCK II-MDR1 and MDCK II-MRP2 cells indicate significant evasion of prodrugs efflux by P-gp and MRP2. Also, permeability studies across Caco-2 cells indicate that the prodrugs are transported by peptide transporters and have increased permeability as compared with the parent drug. Enzymatic stability studies in Caco-2 cell homogenates indicate that the peptide prodrugs are first converted to the ester intermediate (amino acid prodrug V-LVR) and finally to the parent drug [63]. The use of peptides prodrugs of **26** resulted in targeted delivery via peptide transporters, significant evasion of efflux and prevention of CYP3A4 mediated metabolism [64].

#### 3.1.3. Hepatitis Virus (B and C)

**Figure 14:**
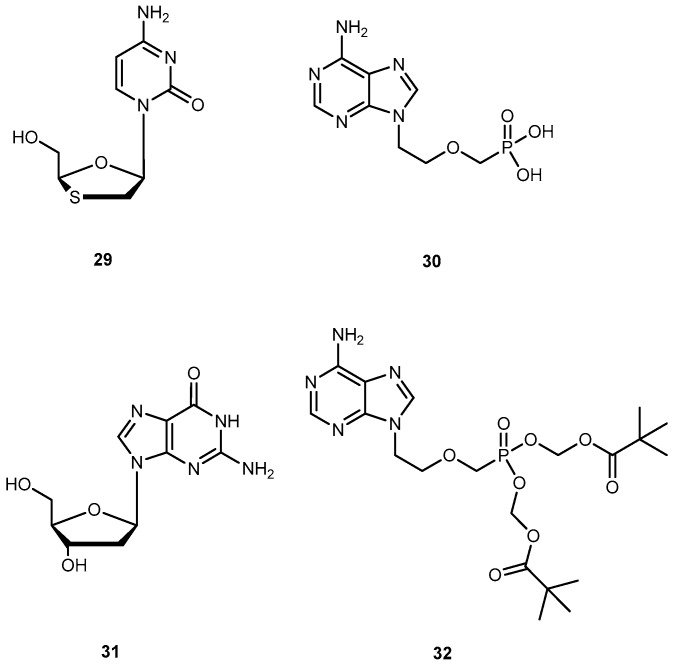
Chemical structures of drugs, lamivudine (**29**), adefovir (**30**), entecavir (**31**) and PMEA (**32**), used against chronic infection of hepatitis B virus.

Hepatitis B virus can cause both acute and chronic infections. Only a few drugs are currently approved by the FDA for the treatment of chronic HBV infection, namely lamivudine (**29**), adefovir (**30**), and entecavir (**31**) (Figure 14). One of major drawbacks of **29** is the low sustained response rate, and drug resistance which affects its efficacy [65,66]. **30**, an ester prodrug of 9[2-(phosphonemetoxy)ethyl]adenine (PMEA, **32**, Figure 14) has a potent in vitro and in vivo activity against HBV, in particular, the ability to suppress replication of HBV resistance to other drugs [66]. However, it presents several undesirable effects, such as dose-limiting nephrotoxicity and its potential toxic metabolites [67].

**Figure 15:**
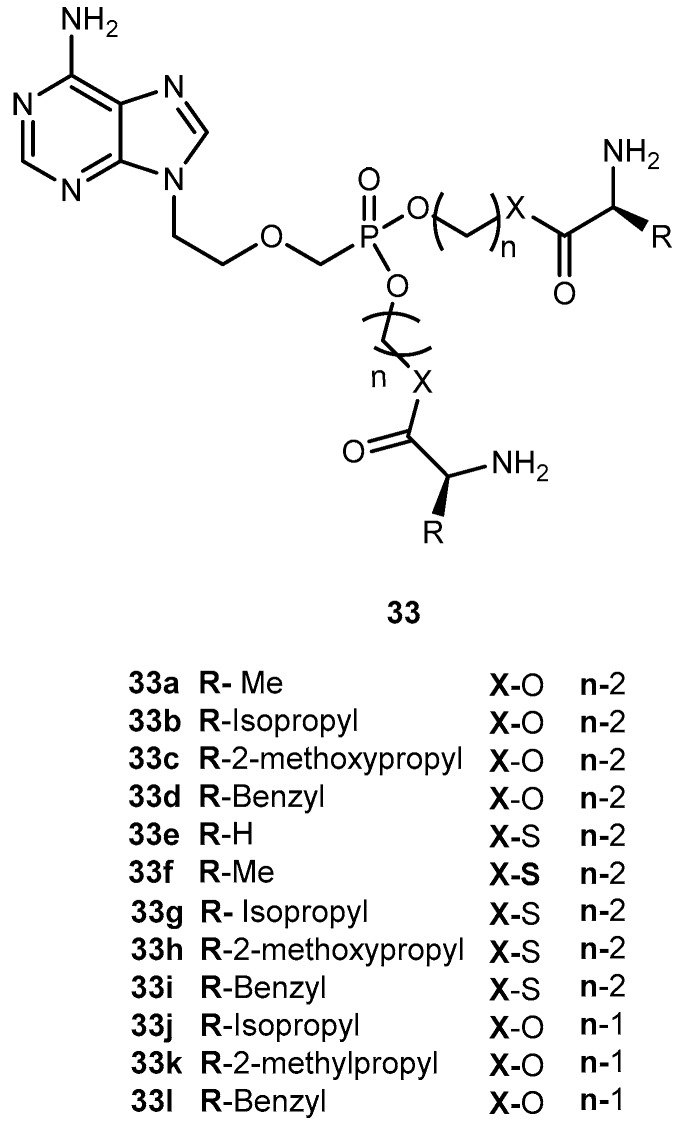
Structures of amino acid ester prodrugs of **32**.

In order to improve pharmacological properties of **32** as anti-HBV, bioavailability, and decreased cytotoxicity, Fu et al. synthesized a series of novel bis(*L*-amino acid) ester prodrugs of **32** depicted in Figure 15. The anti-HBV activity was evaluated in HepG 2.2.1.5 cells [68].

The amino acid prodrugs **33c**, **33d**, **33h**, **33i**, **33j** and **33k** demonstrated to be highly active against HBV and have a higher selective index than the parent drug **32**. In comparison to **29** and **30** and all prodrugs tested (with exception of compound **33e**) had much lower activities. Compound **33c**, which was found to be the most potent, was five times more potent than **30**. In addition, selective index value was 60 and 24 times higher than **30** and **29**, respectively. Moreover, in vitro stability studies showed that compound **33c** was relatively more stable than **30** with a half-life of 270 min. Using *L*-amino acid and ester strategy it was possible to increase activity, selectivity and stability of compounds, thus, its potential should be considered in the acyclic nucleoside phosphonate prodrug design [68].

**Figure 16:**
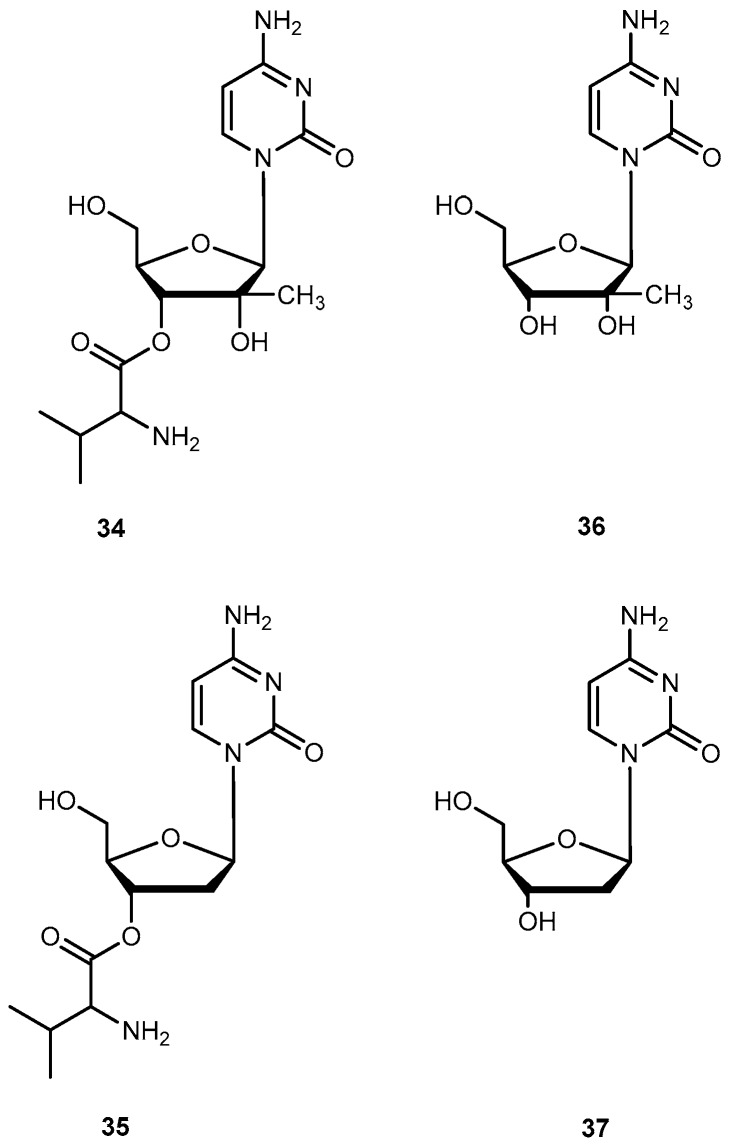
Ester prodrugs **34**, **35** of **36** and **37** respectively.

Valopicitabine (**34**) and valtorcitabine (**35**) are valine ester prodrugs of 2′-C-methylcytidine (**36**) and 2′-deoxy-β-l-cytidine (**37**) respectively (Figure 16). The **37** is a 2′-modified nucleoside analog with specific activity against Hepatitis C virus (HCV) by inhibiting HCV RNA replication. Both drugs are readily phosphorylated in cells to the corresponding active triphosphate forms. Similar to other nucleosides analogs, **36** and **37** also have low oral availability. Due to previously successful applications of valine esters, **34** and **35** were synthesized in order to improve oral bioavailability of the parent drug [69,70]. In fact, **34** has a remarkable bioavailability of 84% in monkeys in comparison to 16% demonstrated by **37** [69]. This enhancement may potentially be attributed to the involvement of PepT1 transport.

Initially, the 3′,5′-divaline ester of **37** was selected as a clinical candidate due to its ease of synthesis. However, it was replaced by the **35** during phase I/II clinical studies because **34** was more stable and had similar bioavailability compared to the 3′,5′-divaline ester in humans. Nowadays, prodrug **35** is in development for the treatment of HBV [71]. Pharmacokinetic studies of **34** showed an oral bioavailability of 34% in rats and 68% in humans [70,72] and was readily and extensively converted to the parent drug after oral doses in patients with HCV infection [73]. Due to these encouraging results, **34** is currently in clinical trials for the treatment of HCV infection [71].

### 3.2. Anticancer

Anticancer drugs are primarily cytotoxic agents and exert their antitumor activity by interfering with some aspects of DNA replication, repair, translation or cell division. However, they do not destroy only tumor cells while sparing the normal cells, which leads to severe adverse effects [74]. Therefore, the main goal of using prodrug strategy of anticancer drugs is reduced their toxicity. As a mean to reduce the toxic effects of these agents, prodrugs designed for selective activation in target tissues are by far the most efficient and attractive option [74,75]. In order to an effective targeting, it is necessary that an enzyme or transporter that is exclusively or preferentially expressed in target tissue which constitutes a major challenge. Nonetheless, several prodrugs strategies have been also employed to improve solubility, transport and pharmacokinetic properties [75]. Since there is an excellent published review on anticancer agent prodrugs [74], here we summarize the anticancer prodrugs using amino acids or peptides as promoieties.

The successful results obtained for **1a** and valganciclovir, amino acid ester prodrugs of **1** and **2**, respectively, has prompted the potential of amino acids as promoieties for other agents. This success has been attributed to their enhanced intestinal transport via oligopeptide transporters. In fact, amino acid ester prodrugs significantly improve the cellular uptake of the parent drugs via peptide transport mechanism, though there is no peptide bond in their structures [31]. The fact that some epithelial cancer cells are rich in these transporters allows their use for the delivery of peptidomimetic anticancer agents [4,76,77,78].

#### 3.2.1. Hepatocellular, Pancreatic Carcinoma and Colon-Rectal Cancer

**Figure 17:**
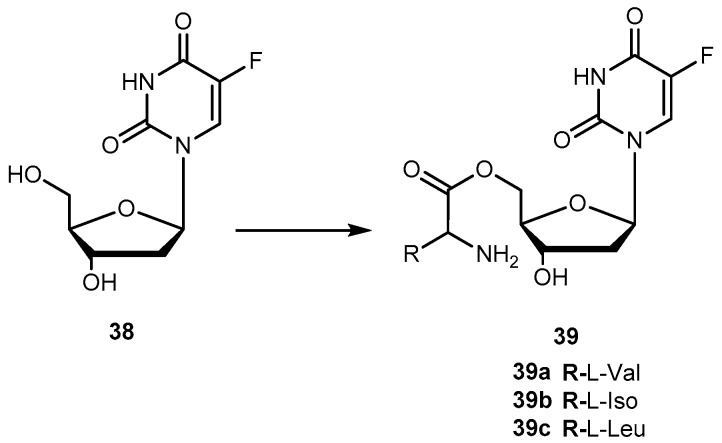
Structures of amino acid ester prodrugs of floxuridine (**38**).

There are in umerous studies of amino acid derivatives of floxuridine (**38**, Figure 17) [4] and gemcitabine [79], both clinically effective anticancer agents, concerning the activation of the prodrug. Unlike the desired rapid activation required for **1a**, extensive intestinal activation of **38** and gemcitabine prodrugs would lead to severe intestinal toxicity. In this regard, several studies have demonstrated that amino acid ester prodrugs (**39a**–**c**, Figure 17) provided resistance to deamination of gemcitabine [79] and to cleavage of **38** [80,81], respectively.

The studies developed with the amino acid esters of **38** were consistent with previous findings. The prodrug **39a** was the most efficiently transported, exhibiting the highest PEPT1-mediated transport and permeability across Caco-2 monolayers. The length and stereochemistry of the amino acid moiety side chain influence the transport efficiency of prodrugs of **38**. The slightly more branched isoleucyl side chain reduced transport of these prodrugs to half, but the branching at γ carbon (as in leucine) side chain highly decreases this transport. The permeability of **39a**–**c** across Caco-2 monolayers was significantly higher than that of the parent drug and also reflected a profound promoiety dependency. Thus, the permeability of the **39a** was roughly 2- and 5-fold higher than the permeability of **39b** and **39c**, respectively [80,81].

The metabolic conversion of **38** to 5-fluorouracil (5-FU) following systemic delivery decreases its therapeutic efficacy. The mechanism of action of **38** and its major metabolite is well understood. The toxicity associated to 5-FU is mostly caused by its incorporation into RNA. Unlike to its metabolite, **38** is specifically incorporated into DNA leading the minimization of adverse effects. Of note that **38** has shown to inhibit cell proliferation 10- to 100-fold more than 5-FU. However, **38** is rapidly converted to 5-FU in many tissues, including the liver, by the enzyme thymidine phosphorylase [82]. Consequently, to maintain the clinical efficacy, higher doses of **38** are required which increase toxicity. In order to counteract this transformation, it is necessary development prodrugs that were protected against the action of this enzyme in order to enhance parental drugs’ efficacy at low doses and reduce its toxicity. In fact, amino acid ester prodrugs were resistant to glycosidic bond cleavage by thymidine phosphorylase. This evidence reinforces the notion that the modification of one or both free hydroxyl groups on the sugar moiety provides protection from glycosidic bond cleavages. The rate of conversion of the prodrugs to the parent drug after transport would influence disposition of **38** and therapeutic action [81]. As previously reported by Vig et al., the structure, stereochemistry, and site of esterification of the amino acid promoiety affect the rates of activation of prodrugs of **38**. Therefore, relating the prodrugs structure with hydrolysis rate it is possible development a prodrug with the desired half-life [78,81].

The roughly 5- to 12-fold higher activity in Caco-2 cell homogenates compared with pH 7.4 buffer suggests the predominance of enzymatic bioconversion of the prodrugs. The results obtained for **39c** indicate that it would not be a suitable candidate. In comparison, **39b** is enzymatically more stable than **39a** and the reference drug **1a** [81]. The combined results of the in vitro studies suggest that isoleucyl monoesters of **38** may be promising candidates for improving oral bioavailability of this drug in vivo. Hypothetically, the prodrugs administered orally could improve intestinal uptake of **38** as well as shield it from unwanted degradation [81]. Tsume and co-workers described the synthesis, characterization, and stability of dipeptide monoesters prodrugs of **38**. Various dipeptides and peptidomimetics have been tested to characterize the hPEPT1 transporter and improve its affinity, and mono-amino acid ester prodrugs have been evaluated as hPEPT1 substrates [79,80,81]. Based on those reports, six amino acids were chosen to be *N*-terminal amino acids of the dipeptide, and other three were chosen to be paired with those six amino acids to test the hypothesis that molecular sizes may structurally affect its ester bond stability [82]. From this study, it was possible to realize that dipeptide prodrugs appeared to be less stable in pH 7.4 buffers than the corresponding mono-amino acid ester prodrugs. Since no mono-amino acid ester prodrug degradation products was detected, it is quite likely that the dipeptide monoester prodrugs degrade through parallel pathways, as previously suggested for the anti-viral dipeptide prodrugs [82].

**Figure 18:**
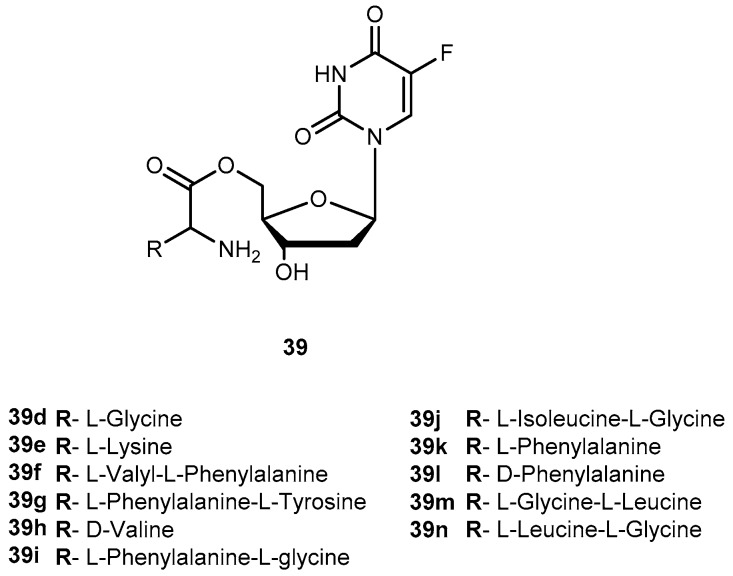
Structures of amino acid and dipeptide ester prodrugs of **38**.

In order to evaluate the advantages of amino acid/dipeptide monoester prodrugs for cancer treatment, Tsume and co-workers designed a series of 5′-monoester prodrugs of **38** (Figure 18) and assessed their uptake and cytotoxic effects in a secondary cancer cell monolayer following permeation across a primary cancer cell monolayer.

Generally, all prodrugs exhibited greater permeation across the first pancreatic cancer cells monolayer, Capan-2, than the parent drug. The results of uptake and growth inhibition on the second layer indicated that amino acid/dipeptide monoester prodrugs (**39d**–**i** and **39j**–**n**), that remain as intact prodrugs following permeation across the first Capan-2 monolayer, provide an enhanced cell penetration and cytotoxic effects on a secondary layer than their metabolites or **38**. It was possible to establish a correlation between uptake and growth inhibition in the second monolayer with intact prodrug permeating the first monolayer. This suggests that permeability and enzymatic stability are essential for the sustained action of prodrugs in deeper layers of tumors. Therefore, stable prodrugs might enhance delivery of the active drug to inner layers of tumor cells compared to parent drug or metabolized prodrugs, being more efficient on cancer treatment [83].

More recently, was reported the potential activation of enzyme cathepsin D by 5′-amino acid/dipeptide monoester prodrugs of **38** in Capan-2 cells, and the feasibility of specific activation of prodrugs. Cathepsin D might be a good candidate as the target enzyme for prodrug activation due its regulation and redistribution to other cellular compartments in tumor cells and its substrate specificity. The results of stability studies with the presence of enzyme inhibition indicate there are particular enzymes activating **39g** and **39i** as cathepsin B and D that significantly activated these prodrugs to produce **38**. For tumors that express large amounts of cathepsin, it is likely that a substantial proportion of **39g** is hydrolyzed by cathepsin D and, therefore, this enzyme has the ability to activate the dipeptide monoester prodrug of **38**, and has the potential to be a target enzyme for prodrug activation in tumors [84].

**Figure 19:**
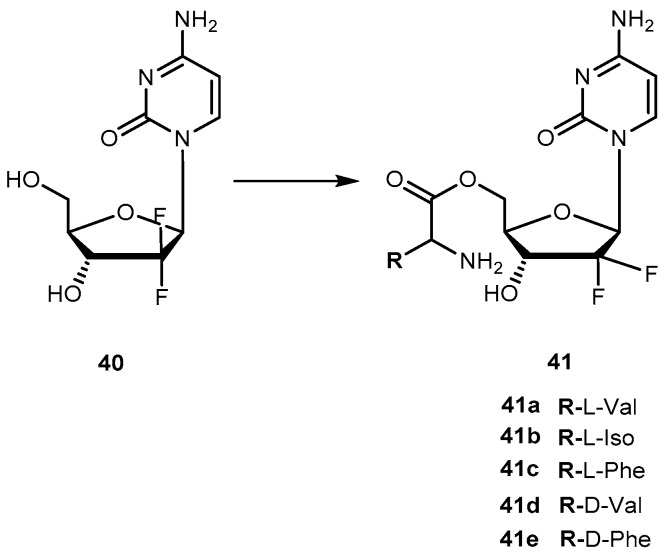
Structures of amino acid ester prodrugs of **40**.

Although gemcitabine (**40**, Figure 19) is clinically effective in the treatment of advanced or metastatic pancreatic cancer, it also exhibits several side effects that are attributed to its inability to distinguish between normal and target cells. It is known that **40** exerts its anti-proliferative activity via multiple mechanisms of action. It is initially phosphorylated intracellularly by deoxycytidine kinase and subsequently by nucleotide kinases to its active metabolites, diphosphate and triphosphate **40**. The influence of **40** on DNA synthesis has been strongly correlated with its triphosphate metabolite intracellular concentration. However, extensive degradation of **40** by cytidine deaminases to an inactive metabolite decreases its activity [79,85]. To overcome these disadvantages, a novel amino acid ester prodrugs of **40** was developed and their affinity to oligopeptide transporters (hPEPT1) that are overexpressed in the gastrointestinal tract and also, in tumoral cells was evaluated [78,79]. To design the amino acid ester prodrugs the aliphatic amino acids *L*-valine, *D*-valine, and *L*-isoleucine, as well as the aromatic amino acids *L*-phenylalanine and *D*-phenylalanine was used (Figure 19). Similarly to previous studies, authors preferred 5′-monoester substitutions and the *L*-configuration of amino acids. In fact, the results were consistent with previously mentioned findings of prodrugs developed using this strategy. Generally, all prodrugs of **40** have greater affinity to the oligopeptide transporter than the parent drug [79].

The chemical stability and rapid enzymatic bioconversion characteristic of **41a** suggest its potential in enhancing oral absorption of the parent drug. On the other hand, **41b** showed a slow bioconversion in Caco-2 cells and in human plasma, as well as an unusual resistance to cytidine deaminase deactivation. Thus, a longer systemic circulation half-life of prodrugs may promote an accurate targeting of cells that overexpress hPEPT-1 transporter [79]. In addition to their affinity to hPEPT1, several properties of **41a**, **41b**, **41d** and **41e** were evaluated as (1) their chemical stability in different media buffers; (2) resistance to glycosidic bond metabolism; (3) enzymatic activation; (4) permeability in Caco-2 cells; (5) mouse intestinal metabolism and (6) anti-proliferation activity in cancer cells [79]. 

Prodrugs containing *D*-configuration amino acids (**41d** and **e**) were enzymatically more stable than *L*-configuration ones (**41a** and **b**). The activation of all prodrugs was 1.3-17.6-fold faster in cancer cell homogenates than hydrolysis in a buffer suggesting an enzymatic activation. This activation on prodrugs containing *D*-configuration of amino acid in cell homogenates was 2.2-10.9-fold slower compared with those with *L*-configuration. All prodrugs exhibited increased resistance to glycosidic bond metabolism by thymidine phosphorylase compared to parent drug and also showed superior effective permeability in mice jejunum than **40**. More importantly, the high plasma concentration of *D*-amino acid prodrugs was observed in more than one of *L*-configuration prodrugs of **40**. In general, all prodrugs exhibited higher permeability and uptake than their parent drug. Cell proliferation assay in ASPC-1 pancreatic ductal cell line indicated that prodrugs were more active than their parent drugs. In addition, the transport and enzymatic profiles of **41d** and **41e**, suggest their potential to increase oral uptake, delay enzymatic bioconversion and enhance uptake and cytotoxic activity in cancer cells [86].

More recently, novel derivatives of **40** were developed using cell-penetrating dipeptides (CPP) in order to facilitate intracellular delivery. In this case, the novel drugs were tested on three cancer cell lines as Caco-2 (Caucasian colon adenocarcinoma cell line), MKN-28 (human gastric epithelium) and HT-29 (colon adenocarcinoma). The results revealed a significant increase of antiproliferative activity of novel CPP-drug conjugates on three cell lines in vitro. In addition, their half-lives were also increased to approximately 9.6 days and 42 h. Taken together, these results demonstrated that conjugation of **40** with CPP might be a valuable strategy for the development of novel prodrugs for an accurate delivery on cancer cells, improving the efficacy of parent drug and alleviate the adverse effects induced on patients [87].

Both strategies used for development of prodrugs of **40** might have great value to develop a novel oral dosage form for anti-cancer agents, not only reducing drug toxicity but also improving targeting of cancer cells. Therefore, the quality of life for the cancer patients could be significantly improved.

**Figure 20:**
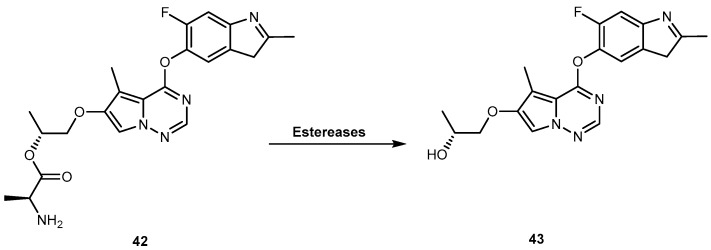
Bioconversion of brivanib alaninate (**42**) to brivanib (**43**) by esterases.

Brivanib alaninate (BMS-582664, **42**, Figure 20) is an investigational amino acid ester prodrug of brivanib (BMS-540215, **43**, Figure 20), a selective dual inhibitor of vascular endothelial growth factor receptor 2 (VEGFR-2) and fibroblast growth factor receptor 1 (FGFR-1). Since 2011, **42** is in phase III clinical trials for the treatment of hepatocellular carcinoma and colon-rectal cancer [88]. It was designed in order to improve the very low aqueous solubility of **43** (<1 μg/mL, at pH 6.5), which contributes to its solubility/dissolution rate-limited oral bioavailability, particularly at high doses. **42** has very high aqueous solubility (73 mg/mL at pH 5.8), which results in a remarkably improved oral bioavailability of **43** up to 52–97% in several animal models [89,90]. In fact, **42** is rapidly and completely converted to **43**, by various esterases (Figure 20), increasing exposure to the parent drug. Therefore, **42** offers an excellent way to deliver **43**, orally [91].

**Figure 21:**
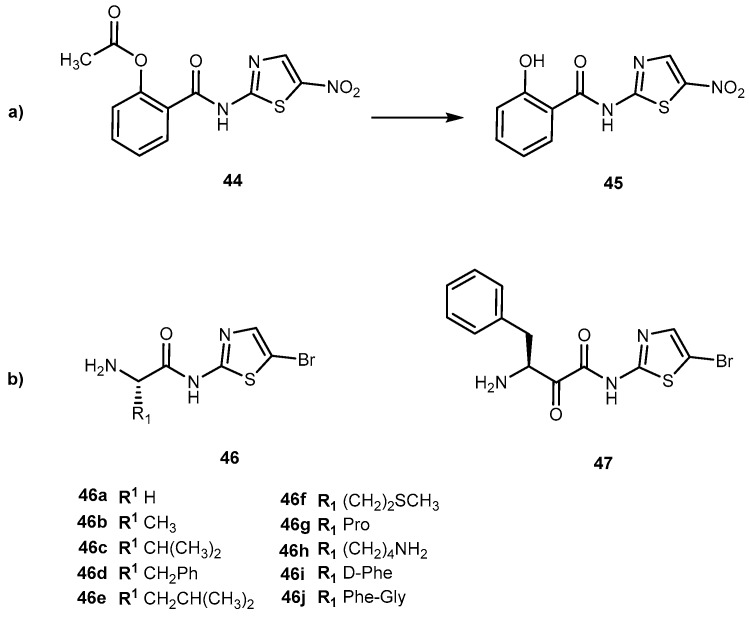
(**a**) Chemical structure of nitazoxanide (**44**) and its major active metabolite tizoxanide (**45**); (**b**) Bromothialozes derivatives with an amino acid moiety (**46a**–**j**) and dipeptide.

Recently, novel derivatives of bromothiazole derivatives with amino acid moieties and a core of nitazoxanide (**44**, Figure 21a) were synthesized and their anticancer, antiproliferative and cytotoxicity effects evaluated [92]. Generally, **44** is used as an anthelminthic agent, mostly used against infections caused by protozoa and helminths [93]. However, the combination of **44** with irinotecan was identified as a potential candidate for the treatment of colon-rectal cancer [94]. **44** acts as a prodrug itself that is deacetylated in the gastrointestinal tract to its active metabolite tizoxanide (**45**, Figure 21). With this in mind, authors developed novel bromothiazoles using 2-amino-5-bromothiazole as starting material and introduced α-amino acids with different nature of side-chain (from one hydrogen to a large heterocyclic group; *L*- and *D*-form) attached to their α-carbon (Figure 21b).

Additionally, a dipeptide was also developed. The novel derivatives presented a significant and concentration-dependent anti-proliferative effect. They were able to reduce ^3^H-thymidine incorporation by more than 80%. When compared to drugs, butyrate, compounds **46c**, **46d**, **46g**–**i** and **47** has a similar cytotoxic effect of butyrate. In comparison to **44**, **46a**, **46c**, **46f** and **46g** have a better performance, particularly **46f** which showed an enhancement of 22% [92]. This compound has a methionine (Met) moiety which is relevant since cancer cells have a unique metabolic addition to in contrast with normal cells. Moreover, *S*-adenosylmethionine metabolism is dependent on Met availability in cancer cells [95]. This might suggest that drugs that included Met in their constitution might be suitable to target cancer cells.

#### 3.2.2. Brain Cancer

**Figure 22:**
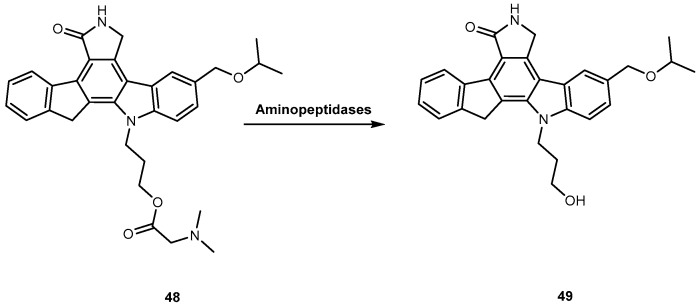
Bioconversion of amino acid ester prodrug **48** to its parent drug **49** by aminopeptidases (Reproduced from [4]).

CEP 7055 (**48**, Figure 22) is a dimethyl glycine ester of CEP 5214 (**49**, Figure 22), a pan-inhibitor of VEGFR tyrosine kinases with antitumor activity [96]. The latter has a very low aqueous solubility, which might be responsible for its poor oral bioavailability [97]. Notwithstanding, the use amino acid prodrug strategy to produce **48** increased solubility up to 4000-fold (comparatively to **49**) and improved bioavailability between 15–20%. The produg **48** is hydrolyzed to the active metabolite (**49**), during the absorption of the enterocytes by aminopeptidases (Figure 22). Currently, **48** has advanced to clinical trials as a chemotherapeutic agent for glioblastoma and colon cancer in combined therapy [96,98].

**Figure 23:**
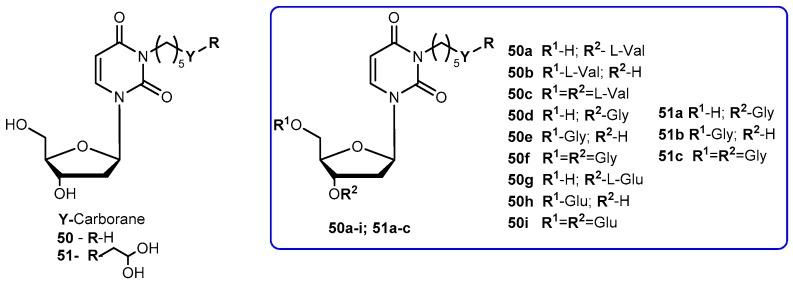
Chemical structures of 3-carbonyl thymidine analogs, N5 (**50**) and N5-2-OH (**51**) and its amino acids prodrugs **50a**–**i** and **51a**–**c**.

3-Carbonyl thymidine analogs (3-CTAs), such as N5 (**50**, Figure 23) and N5-2-OH (**51**, Figure 23), have been developed for Boron Neutron Capture Therapy (BNCT) for high-grade brain tumors as glioblastoma multiforme (GBM) [99]. BNTC is a binary cancer treatment modality that is based on irradiation of boron-10 (^10^B), a stable isotope, with low energy neutrons [100].

Despite the potential interest in **50** and **51** for BCNT, these compounds present a major drawback: both are very lipophilic due to the presence of the carborane cluster and the absence of any potential groups that can be ionized under physiological conditions. In order to improve this, Hasabelnaby and his co-workers synthesized amount water-soluble amino acid esters prodrugs of **50** and **51** using as promoities *L*-Val, *L*-Glu, and glycine (Figure 23). These prodrugs were prepared and stored as hydrochloride salts [101].

Water solubilities of synthesized prodrugs were evaluated in PBS at different pH (5.6 and 7.4) demonstrating that those improved 48–6600 times in comparison with parent drugs. Additionally, their stability was evaluated in different media: PBS at pH 7.4, bovine serum, and bovine cerebrospinal fluid (CSF). The solubilities for all prodrugs are significantly better than parent drugs and appeared to be suitable for injection formulations. The rate of the hydrolysis in all incubation media depended primarily on the amino acid promoiety and, to a lesser extent, to the site of esterification at the deoxyribose portion of 3-CTAs. Generally, glycine esters (**50d**–**f**) and **51a** were 20–25 times more sensitive to chemical hydrolysis than the correspondent glutamate **50g**–**i** and valine ester **51a**–**c**. These results may be explained by the presence of the small glycine promoiety, which sterically does not interfere with the nucleophilic hydrolytic attack at the ester bond. Comparing the hydrolysis rate of the amino acid ester prodrugs in other media: it was overall higher in bovine CSF comparatively to PBS and somewhat lower than in bovine serum. Despite a low concentration is present in the CSF, enzymatic degradation may be possible causing an increased rate of the hydrolysis in this fluid compared with PBS. In case of **50h**, its significant rate of hydrolysis in CSF may be related to the presence of glutamate-specific enzymes, since glutamate is the most common neurotransmitters in the brain. Therefore, its presence might be crucial for developing prodrugs for brain diseases. The rapid hydrolysis in CSF with stability in PBS at pH 7.4, make compounds **50e**, **50h** and **51b** the most promising candidates for preclinical BNCT studies [101].

**Figure 24:**
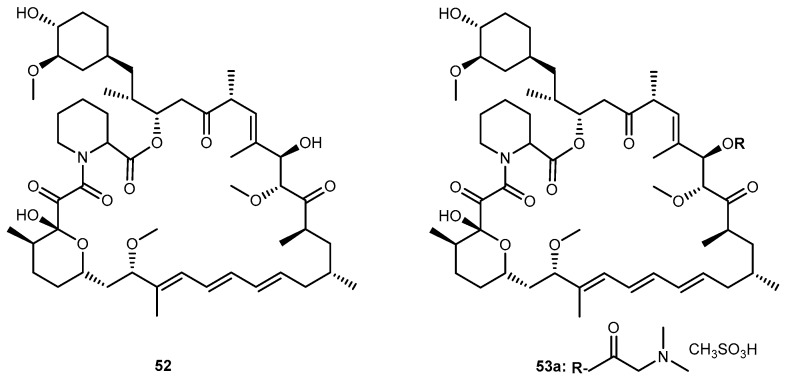
Structure of rapamycin (**52**) and its glycinate derivative (**53a**).

Rapamycin (**52**, Figure 24), an immunosuppressant and chemotherapeutical drug used to prevent rejection after organ transplantation, has low aqueous solubility and poor oral bioavailability. Rapamycin-28-*N*,*N*-dimethylglycinate methanesulfonate salt (**53**, Figure 24) was synthesized as a potential water-soluble prodrug to facilitate parenteral administration of the antineoplastic macrolide **52** [102].

Effective therapeutic treatment regimens of **52** appear to be considerably less toxic to mice than other anticancer agents. The apparent penetration of **52** through the blood-barrier brain (BBB) was evident by high activity against intracranially implanted tumors, including U-251 human glioma [102].

After the introduction of dimethylglycinate methanesulfonate salt into **52**, it was noted a persistent for 5–12 h in mice treated with a dose ranging 10 to 100 mg/kg. The disposition of **53** exhibited an atypical dose-dependency that appears to originate from the saturable binding of the compound to the plasma proteins while binding to tissues remains linear. Although systematically circulating prodrug may be subject to elimination by a variety of pathways, **53** effectively served as a slow-release delivery system for the parent compound. Single doses ranging 10 to 100 mg/kg given by bolus intravenous injection provided a plasma concentration of prodrug **53a** that were sustained at a near-peak level for approximately 8 h and remained above 0.1 μm for 48 h. These observations imply the possibility of maintaining therapeutic plasma levels of the drug on a more convenient dosing regimen than a continuous infusion schedule [103]. Considering this, the demonstration of activity of **53** against in vivo brain tumors models should be pursued for the continuous development of novel and effective prodrugs for the treatment of brain neoplasms.

#### 3.2.3. Human Lung Carcinoma

**Figure 25:**
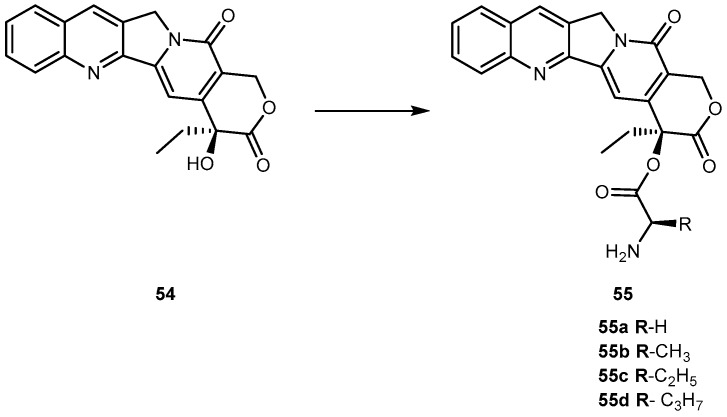
Structures of prodrugs of **54**.

Camptothecin (**54**, Figure 25) a cytotoxic quinoline alkaloid, is a potent anticancer agent that inhibits both DNA and RNA synthesis. The lactone form of **54** (i.e., active form) is responsible for its anticancer activity [104,105].

Nonetheless, there are two major drawbacks of **54** that have made it less attractive for clinical use. First, **54** has unfavorable physical-chemical properties, and second, it has severe clinical toxicities. Moreover, it has a very poor aqueous solubility making it difficult to formulate. Moreover, under physiological conditions it is rapidly hydrolyzed to the carboxylate form. These carboxylate forms are known to induce severe cumulative hematological toxicity, diarrhea, and chemical or hemorrhagic cystitis [106]. With the intent to identify a prodrug of **54** with optimal release and cytotoxicity properties for immobilization on a passively targeted microparticle delivery system, four α-amino acid ester prodrugs of **54** were synthesized with increasing aliphatic chain length, glycine **55a**, alanine **55b**, aminobutyric acid **55c** and norvaline **55d** (Figure 24) and assessed for human lung carcinoma.

Prodrug reconversion was studied at different pH 6.6, 7.0 and 7.4, corresponding to tumor, lung and extracellular/physiological pH, respectively. **55c** and **55d** present longer release profiles of parent drug than **55a** and **55b**, at the three studied pH values. The hydrophobicity constant appears to be directly proportional to the half-life of both on the transformation into **54** as well as in **55a**–**d** hydrolysis. This evidence suggests that increasing the length of the side chain of the hydrophobic amino acid would sustain the release of **54**. The increase of prodrug reconversion might be directly related to increasing aliphatic chain lengths due to its effect of steric hindrance. Since **55d** present lower toxicity and sustain release of parent drugs, **55d** should be considered for a passively targeted sustained release lung delivery system [107]. As previously mentioned it is crucial that prodrug should be resistant to hydrolysis in order to achieve a sustained and targeted delivery. It seems that longer aliphatic chains coupled to amino acid moiety might be a very valuable strategy to achieve this goal, turning prodrug more resistant to hydrolysis performed by patient’s enzymes.

#### 3.2.4. Breast Cancer

**Figure 26:**
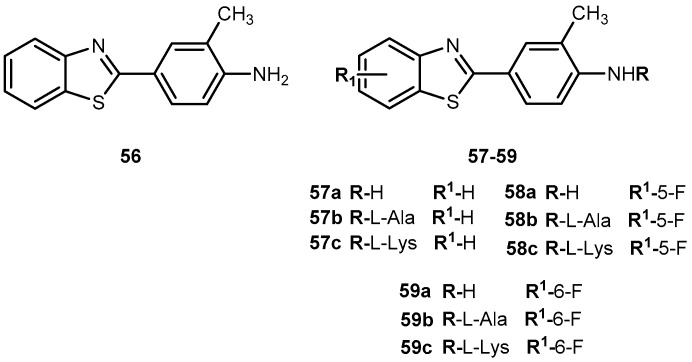
Structure of 2-(4-aminophenyl)benzothiazole **56** and its amino acid prodrugs **57a**–**c**, **58a**–**c**, and **59a**–**c**.

A novel antitumor agent 2-(4-amino-3-methylphenyl)benzothiazole (**56**, Figure 26) was demonstrated to be highly selective and display potent antitumor properties both in vitro and in vivo. Apparently, the induction of CYP1A1-catalyzed biotransformation of **56** is crucial to its antitumor activity [108,109]. The formulation of an aqueous intravenous formulation constitutes a pharmacological challenge, however, it is fundamental to minimize the possibility of first pass deactivating metabolism and improve drug bioavailability [110]. Therefore, novel amino acid ester prodrugs of **56** were developed (Figure 26) and their water and chemical stability were assessed on different animal models (mice, rats, and dogs).

The introduction of alanyl- and lysyl-amide hydrochloride salts could be conjugated to the exocyclic primary amine functions of **56**, increasing water solubility, chemical stability and suitable for sustained release of the parent drug. Indeed, all prodrugs tested undergo through a rapid and quantitative release of the parent drug. In addition, after administration of **58a**–**c** in mice, plasma levels of **58** exceeding 5-fold the concentrations required to elicit 50% growth inhibition (IG_50_), total growth inhibition (TGI). These activities persisted for 4 h and >6 g after a single intravenous infusion of prodrug **58b** and c in MCF-7, T47D and ZR-75 breast cell lines. Of note that potent and selective antitumor activity of **58a** observed in vitro was retained in vivo. In fact, **58c** was able to suppress significantly the growth of MCF-7 breast and IGROV-1 ovarian xenografts in vivo. Intriguingly, only tumor xenografts whose growth were inhibited by **58c** demonstrated inducible CYP1A1. This suggests that CYP1A1 expression might be a biomarker in human tumors for identification of sensitive tumor phenotypes. On another hand, expression of CYP1A1 might lead to hepato or pulmonary toxicity [110]. Therefore, it is necessary to adjust dose administered in order to prevent undesirable lesions.

In view of encouraging preclinical properties achieved by *L*-Lys-amide dihydrochloride salt combined with the superior efficacy and retained selectivity of compound **58**, prodrug **58c** has been selected to undergo Phase I clinical evaluation on 2002, under auspices of the UK Cancer Researcher Campaign [110].

**Figure 27:**
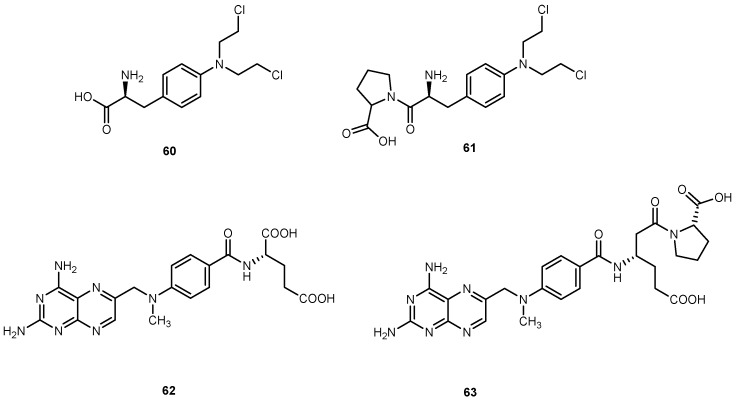
Chemical structures of melphalan (**60**) and methotrexate (**62**) and its proline conjugates (**61** and **63**).

Enzymes are unique in a tissue, or present at a higher concentration compared to other tissues, and therefore can be exploited for site-selective prodrug conversion and hence targeting. Some tumor tissues have been shown to evoke increased prolidase activity compared to normal tissues. With this in mind, novel prodrugs that will be transformed into parent drug by the action of this enzyme could be development [4]. Melphalan (**60**, Figure 27) belongs to the class of antitumor agents with an alkylating and cross-linking action on guanine and possibly other bases of deoxyribonucleic acid (DNA) that result in arresting of all division [111].

Chrzanowski et al. designed conjugates of **60** with proline through imido-bond, which resulted in the formulation of a good substrate for prolidase [112]. A proline drug of methotrexate **62** was also designed as a substrate for prolidase [113]. Both prodrugs showed increased cytotoxicity for breast cancer cell line MDA-MB-213 compared with the parent drug, suggesting that the proline prodrug approach may overcome the resistance associated with parent drug, at least this specific cell line. The increased cytotoxicity was attributed to two mechanisms: (1) proline prodrugs were more effectively transported into MDA-MB-231 cells, likely by transporter-mediated mechanisms; (2) higher prolidase activity in MDA-MB-231 cells than in normal cells, contributing to the effective release of the parent drugs at the site of drug action [112,113].

#### 3.2.5. Melanoma

Different proline prodrugs of **60** were synthesized by direct coupling of a free carboxylic group of **60** to the *N*-terminal imino acid and assessed in melanoma. Although the rationale proposed for the synthesis of proline drugs of **60** involves the targeting of prolidase, the differences in the linkage of the proline moiety to a drug may be crucial, which might affect their activation by prolidase [114].

The antiproliferative activity profiles of **60**, *D*-prophalan and *L*-prophalan in SK-MEL-5 cells (a melanoma cancer cell line with high expression of prolidase) indicate a ~7-fold high rate of activation of derivatives. The relative of GI_50_ for parent drug and proline drugs of **60**, coupled with the stability of the two prodrugs in growth medium indicate that bioconversion of the prodrug to the parent drug may determine their cytotoxic activity in cells [114]. From these reports, it is possible to conclude that prodrugs of **60** that are cleavable by prolidase offer the potential for enhanced selectivity by facilitating cytotoxic activity in cells overexpressing prolidase. Of note that the potential bioactivation of the prodrugs by prolidase expressed in normal tissues was not assessed. In addition, it is necessary to provide information regarding their chemical stability that will be necessary for targeted delivery to melanoma tumor cells [114].

**Figure 28:**
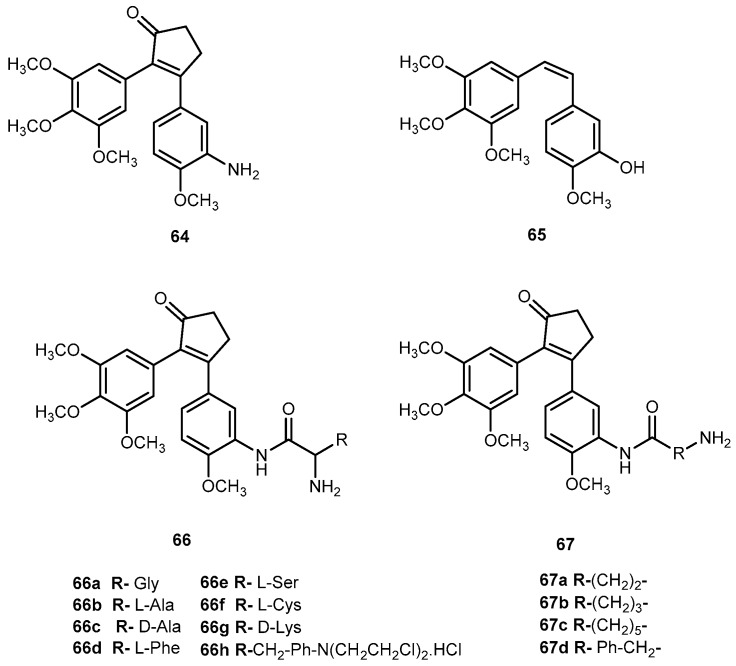
Structures of prodrugs of **64**.

Nam et al., designed a series of prodrugs of antitumor agent 3-[3-(amino-4-methoxy)phenyl-2-(3,4,5-trimethoxyphenyl)cyclopent-2-ene-1-one] (**64**, Figure 28) a novel analog of combretastin A-4, (**65**, Figure 28). The **61** has a very potent cytotoxicity against various tumor cell lines, however, this strong activity was not observed in vivo. This unexpected low tumor activity might be related to its low bioavailability, which is responsible, at least in part, to its poor aqueous solubility [115]. In order to improve this parameter, prodrugs with α-amino acid **66a**–**h**, aliphatic amino acid **67i**–**l**, and phosphoramidate and phosphate derivatives were synthesized (Figure 28) [116].

The introduction of different amino acids and phosphate improved water solubility of the prodrug. In addition, their antitumor activity was also improved with several amino acids prodrugs **66a**, **66b**, **66d**–**f**, **66h**, **66a**, and **67c** exhibiting more potent antitumor activity compared to the parent drug. Cytotoxicity of the prodrugs was determined in two tumor cell lines, B16 (murine melanoma) and HCT 116 (human colon tumor). Most of the amino acid prodrugs of **66**–**67** showed potent cytotoxicity in both tumor cell lines. Nonetheless, it is not clear if prodrugs **66a**–**h** and **67a**–**d** would be cytotoxic *per se* or their toxicity is related to their conversion into parent drug. It should be noted it that none of the prodrugs were significantly toxic in mice [116]. Considering the introduction of amino acids and phosphate group lead to an increase of bioactivity and improvement of water solubility this strategy might be applied to other aromatic amines drugs that have poorly soluble in the aqueous system.

#### 3.2.6. Other Compounds

**Figure 29:**
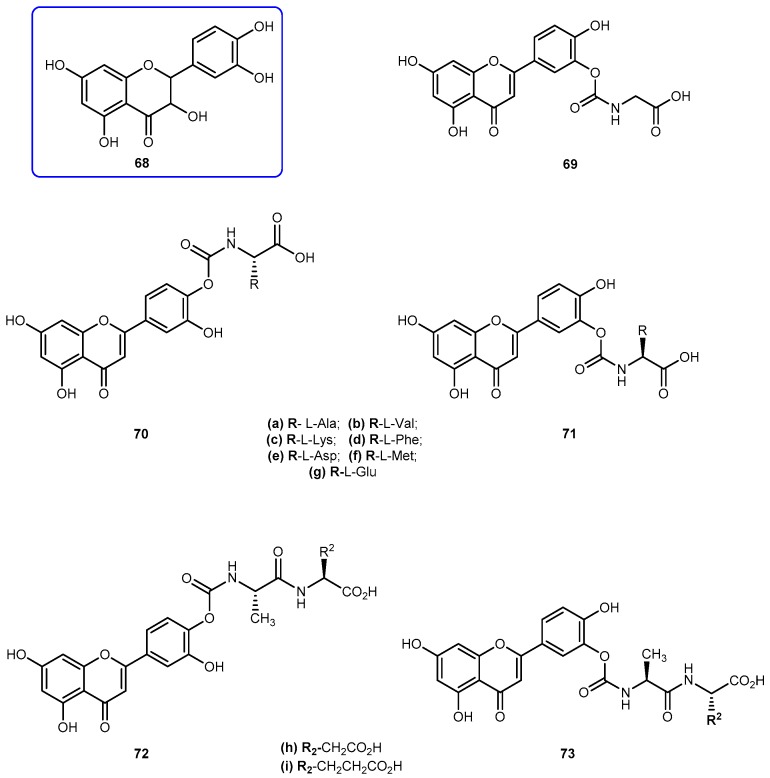
Quercetin (**68**), prodrug QC-12 (**69**) and its amino acids **70**–**71** and dipeptides **72**–**73** conjugates.

Quercetin (3′-4′-3-5-7-tetrahydroxyflavone, **68**, Figure 29) is emerging as anticancer drug candidate and its prodrug QC-12 (**69**, Figure 27) has entered the phase I clinical trials [117]. The **69** is an amino acid prodrug of **68**, where one of the phenol groups of the parent drug is attached to amino acid glycine via carbamate bond which should be cleavage in the bloodstream by hydrolysis to yield the parent drug [118]. Although **68** has many biological activities, the drug is almost water insoluble which requires the use of dimethyl sulfoxide (DMSO) for its use in clinical trials. The introduction of amino acid moiety leading to the formation of **69**, has increased aqueous solubility when compared to the parent drug. The prodrug is activated by cellular hydrolyzing enzymes, however, it is unknown if this activation occurs before or during absorption [119].

The advantage presented by **69** is its high aqueous solubility compared to the parent drug. However, its utility for oral dosage is limited due to the low bioavailability, which presumably results from the fast metabolism and excretion of the prodrug. With intent to improve bioavailability, it was synthesized a novel amino acid/dipeptides conjugates of **69** and assessed their pharmacokinetic properties, including water solubility, stability against chemical or enzymatic hydrolysis and cell permeability across MDCK (Madin-Darby canine kidney) cells. The moieties used to their synthesis were non-polar amino acids (Ala, Val, Phe, Met), positively charged (Lys) and negatively charged (Asp, Glu) (Figure 29) [119].

The prodrugs of **68** showed remarkable increases of 6.8–53.0-fold of water solubilities, increased relative to **68**. In particular, amino acids such as aspartic acid and glutamic acid increased the water solubilities of their corresponding quercetin conjugates **70e/71e** and **70g/71g** by 45.2 and 53.0 folds respectively. The introduction of amino acid chains provided additional solubilities to novel analogs of **69** by blocking hydrolysis on carbamate link. Like **68**, its prodrugs were stable in PBS buffer but susceptible to enzymatic hydrolysis in a cell lysate, which contained various activated hydrolyzing enzymes. Amino acid conjugates of **68** such as **70c/71c** and **70g/71g** showed strong resistance against hydrolysis and consequently has extended half-lives compared with that of **69**. The prodrugs **70e/71e**, **70g/71g** and **72i/73i** also showed significantly increased intestinal permeability at MDCK cells in comparison with parent drug. Due to these results, Kim et al. considered to have identified a novel quercetin-amino acid conjugate **70g/71g**, of which warrant further development as prodrugs of **68** and **69** [119].

**Figure 30:**
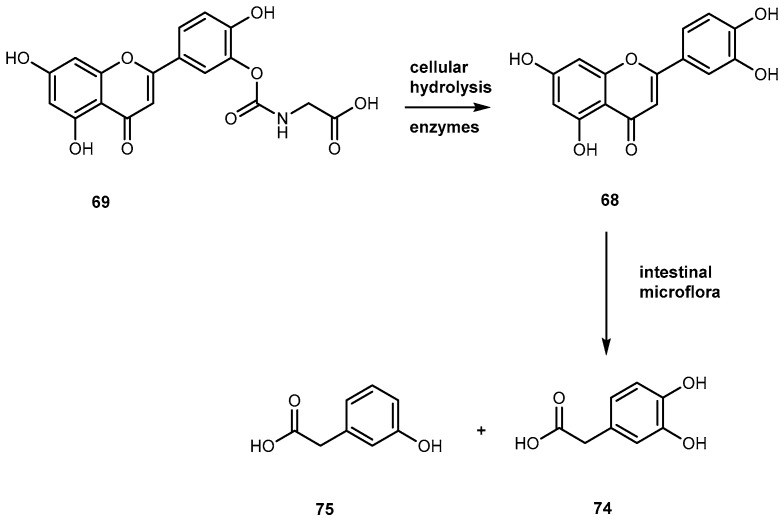
Bioconversion of a carbamate of **69** by cellular hydrolyzing enzymes and the proposed biotransformation to active metabolites, **74** and **75,** by intestinal microflora (Reproduced from [4]).

More recently, it has been proposed that **64** might act as a prodrug itself since it is rapidly transformed into active metabolites, 3-4-dihydroxyphenylacetic acid (**74**) Figure 30) and *m*-hydrophenylacetic acid (**75**) (Figure 30) by intestinal microflora [120].

**Figure 31:**
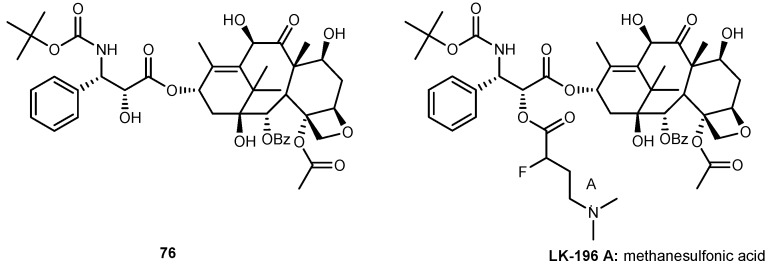
Chemical structure of docetaxel (**76**) and its novel prodrug LK-196.

Docetaxel (**76**, Figure 31), is an important chemotherapeutic drug widely used for the treatment of several types of cancer. Like other drugs, **76** has poor water solubility and consequently has limited clinical application. Moreover, it induces several adverse effects as allergic reactions, neurotoxicity and cardiovascular toxicity [121,122].

In order to improve its antitumor activity and water solubility, Ma et al. developed a novel derivative of **76** by linking an amino acid to the hydroxyl group of C’2 position (Figure 31). Its stability and solubility were assessed on PBS buffer (pH 7.0) and human plasma. Through linkage of amino acid moiety improved its water solubility 200–600 times compared to **76**. Therefore, this new derivative might suitable for injection by preparing a lyophilized powder. In addition, after administration of LK-196 in human plasma, it was demonstrated that the new derivative acts as a prodrug of **76**. This fact might be related to the strong-electron effect caused by fluorine atom in α-position of the amino acid linker. Regard to its antitumor activity, LK-196 significantly inhibited the growth of human prostate tumor PC-3 in nude mice. In fact, LK-196 shown a stronger activity than **76** [123]. Despite these encouraging results, after administration of LK-196 at higher LD_50_, several adverse effects were observed. Nonetheless, its toxicity was not increased when compared to **76** [123]. Apparently, the introduction of the amino acid linker with a fluorine moiety improved the water solubility and stability and did not alter its toxicity. Therefore, this strategy should develop novel derivatives with lower toxicity in vivo.

### 3.3. Antiparasitic Drugs

Parasitic diseases, caused by a diverse spectrum of eukaryotic organisms, represent a major global health problem. Malaria, leishmaniasis, Chaga’s disease and African sleeping sickness (both caused by *Trypanosoma* species) affects hundreds of millions of people worldwide causing millions of deaths annually and present an immense social and economic burden [124]. Most of the current drugs used to treat these diseases, especially the neglected ones, are old and have many limitations, including the emergence of drug resistance [125,126]. Therefore, it is urgent to develop new control tools (vaccines or new molecule-drugs) exhibiting different modes of action by exploring and combining a wide variety of chemical structures or improving pharmacological properties of drugs that are already used.

#### 3.3.1. Trypanosomiasis

**Figure 32:**
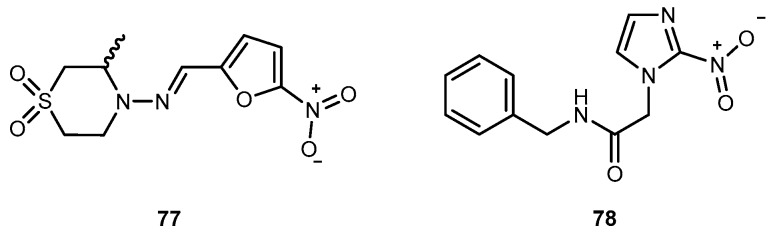
Chemical structures of trypanocidal drugs, nifurtimox (**77**) and benzindazole (**78**).

American trypanosomiasis (Chaga’s disease) represents a serious public health problem in the American continent, where approximately 100 million is at the risk of infection. Current therapy is unsatisfactory and accomplished with the only two available trypanocidal drugs, nifurtimox (**77**) and benzindazole (**78**) (Figure 32) [127] which are only effective in the acute phase of the disease.

**Figure 33:**
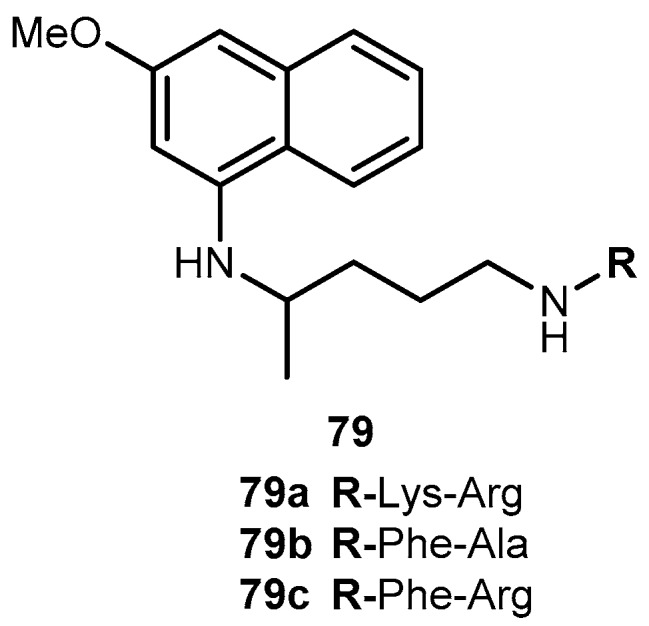
Dipeptide prodrugs of **6** tested against trypanosomiasis.

One of the major challenges in trypanosomiasis chemotherapy is the related efficacy of drugs, they are only effective in the acute phase of the infection. However, early stage of the infection is difficult to diagnose. On the other hand, both drugs have serious adverse side effects. The antimalarial **4**, has also been used in human acute and congenital cases of Chaga’s disease [128]. Chung and co-workers synthesized dipeptide prodrugs of **4** (Figure 33) and evaluated in vitro trypanocidal activity.

Dipeptide prodrugs of **4** (Figure 33) were active against trypomastigotes forms of *Trypanosoma cruzi* (*T. cruzi*) that were present on Rhesus monkey kidney epithelial cells LLC-MK2. It was demonstrated that **79a** was active on *T. cruzi* development inside host cells, probably by interfering in the initial steps of trypomastigote-amastigote transformation. These prodrugs proved to be more active than **79b** and **c**, suggesting that the specific cleavage has an important role in the release of **6**. In addition, it was a good carrier for **4** and has a potential to be used as a spacer group for the development of other prodrugs of **4** against trypanosomiasis [127]. Therefore, **79a** should be considered for in vivo test provide information regarding to its bioavailability and other pharmacological parameters.

**Figure 34:**
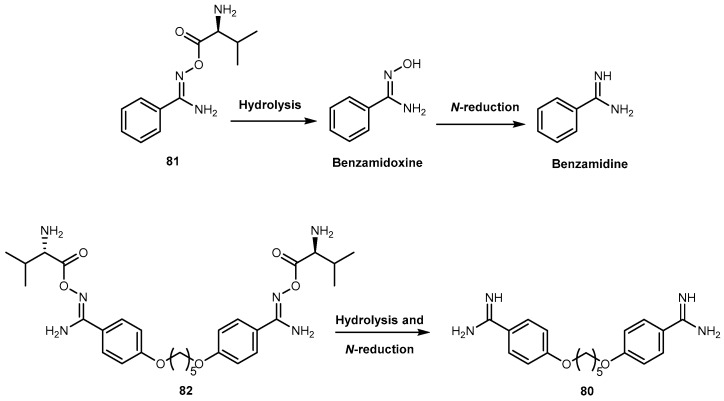
Metabolic activation of *N*-valoxybenzamidine and *N*,*N*′-bis(valoxy)pentamidine (reproduced from [131]).

Pentamidine (**80**, Figure 34) is effective in therapy for the hemolymphatic stage of trypanosomiasis and antimony-resistant leishmaniasis [129]. As an aromatic diamine, pentadimine requires an intravenous or inhalation application. Unfortunately, most of the serious parasitic infections occur in tropical or subtropical countries that usually have poor medical care system. Consequently, this way of application limits the medicinal use of **80** in most regions and emerging need for a derivative of **80** that can be administered orally. 

Moreover, the intravenous application is associated with toxic side effects [130]. Generally, drugs containing amidines has poor oral bioavailability and are often converted into amidoxime prodrugs to overcome low uptake from GI tract. Esterification of amidoxime with amino acids represent a newly developed double prodrug principle. In order to reduce toxicity, Kotthaus and co-workers designed a model compound *N*-valoxybenzamidine (**81**, Figure 34) and investigated it’s biological transformation in vitro and in vivo, with a special interest in oral bioavailability and the tissue distribution after application. Afterwards, the prodrug principle was transferred to *N*,*N*′-bis(valoxy)pentamidine (**82**, Figure 34). Kotthaus and co-workers examined water solubility, stability, in vitro bioactivation, and organ distribution of **81**, including post absorptive conversion to the active metabolite pentamidine in rats [131].

Both **81** and **82** were activated in vitro by all enzyme preparations investigated (i.e., porcine and human subcellular enzyme fractions, hmARC1, hmARC2, and hepatocytes). The activation relies on esterases and mitochondrial amidoxime reducing components (mARC) and is thus independent of P450 enzymes, minimizing the risk for drug-drug interactions and undesired effects. Kotthaus and co-workers results demonstrated the increase of solubility in comparison to the amidoxime prodrugs leading to an excellent oral bioavailability. In addition to absorption by diffusion, the transport by amino acid and peptide transporters in the GI tract is feasible. After oral administration in rats was observed an oral bioavailability of prodrug about 88% was observed **81**. The high bioavailability also excludes the possibility of an ester hydrolysis prior to absorption. Compound **80** entered into the cells of all tissues investigated. This effect is essential for the antiprotozoal effect of **80** considering that the parasites are spread throughout the body. More important, **81** was transformed completely to the parent drug. Also, its solubility was improved over 100-fold in comparison to **80**, diamoxidine and *N*,*N*′-bis(acetoxy)pentamidine. The authors considered that these observations indicate that the development of this new prodrug principle may also valuable for the treatment of the second stage of African sleeping sickness. Furthermore, Kotthaus and co-workers detected active drug **80** in the brain, although only in small amounts. They considered that the development of prodrugs of **80** based on other amino acids might be an interesting strategy leading to prodrugs able to cross the blood-brain barrier more efficiently [131].

#### 3.3.2. Malaria

Malaria is one of the major public health problems in tropical areas. The rapid emergence and spread of chloroquine-resistant *Plasmodium falciparum* (*P. falciparum*) throughout the African continent is the major obstacle to present efforts to control the disease. *P. falciparum* strains are resistant to newer drugs such as mefloquine which might result from the loss of drug activity on the asexual blood forms of the parasite, called blood schizonts, that are responsible for the clinical symptoms of disease [132].

Current, **4** is the drug of choice against malaria since it is active against both of latent liver forms of relapsing malaria caused by *P. vivax* and *P. ovale* and the gametocytes from all species of the parasite causing human malaria [132]. The short plasma half-life of **4** might be related to its rapid oxidative deamination to carboxyprimaquine [133], which is inactive against malaria. Also, **4** induces oxidation of oxyhemoglobin to methemoglobin leading to a considerable blood toxicity [134]. In order to improve some limitations of **4**, several prodrugs have been developed.

**Figure 35:**
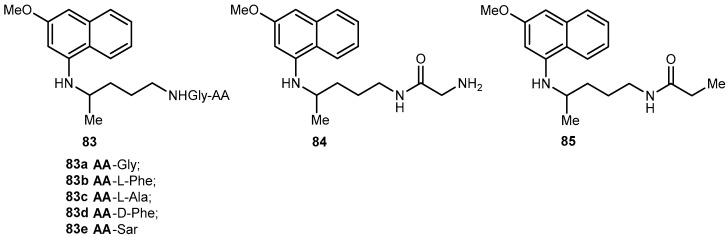
Dipeptides derivatives of **6** synthesized by Portela et al. [37].

Portela and co-workers developed dipeptide derivatives of **4** to (i) evaluate the gametocytocidal activity of novel dipeptides derivatives **83**–**85** of **4** (Figure 35) and their potential as transmission-block antimalarials; (ii) compare the side effects of chains containing natural and non-natural amino acids on the gametocytocidal activity and (iii) to assess the effect of aliphatic side-chain acylation on the formation of carboxyprimaquine in rat liver homogenates [37].

Equal roles of CYP450, monoamine oxidase (MAO) and aldehyde dehydrogenase in the conversion of **4** into carboxyprimaquine, rat liver homogenates were selected to study the metabolism of derivatives **83**. Simultaneous formation of **4** and **84** was observed with compounds **83a**–**c**. In contrast, derivatives **83d**–**e** were stable in the incubation mixtures and their concentrations remaining constant for at least 3 h. Under the same conditions, **84** failed to regenerate the parent drug [37]. Thus, it was considered that acylation of the aliphatic side-chain protects the terminal amino group against oxidative deamination, thereby preventing the formation of carboxyprimaquine [37].

The mefloquine-resistant *P. berghei* ANKA 25R/10 strain, *Balb c* mice, and *Anopheles stephensi* mosquitoes were used throughout the course of the assay for sporogonic development. The criteria used to assess the antimalarial activity of each compound were: (i) the minimal effective dose that prevents the appearance of oocysts in the midgut of mosquitoes; (ii) the percentage of mosquitoes with oocysts; (iii) the mean number of oocysts per injected mosquito; and (iv) % of mosquitoes with sporozoites in their salivary glands. All the derivatives of **83** were able to prevent the development of the sporogonic cycles of *P. berghei* in *A. stephensi* mosquitoes at dose levels of 15 and 7.5 mg/kg. Most likely, the gametocytocidal activity of derivatives of **83** is not related to their rate of peptidase-catalyzed hydrolysis to **4**. The derivatives **83d**–**e** which are not hydrolyzed by peptidases display an intrinsic activity. In contrast, *N*-acetyl-PQ **84**, which lacks a terminal basic amino group, did not present a gametocytocidal activity. Carboxyprimaquine, which itself lacks a terminal group, is also inactive, implying that the presence of a terminal amino group is a major structural requirement for the gametocytocidal activity. Thus, the acylation of the primary amino group at C’-4 with dipeptides containing natural and non-natural amino acids, is a new approach to preventing the oxidative deamination of **4**. Also, they considered this may be useful to the design of new antimalarials based compounds that are rapidly metabolized [37].

**Figure 36:**
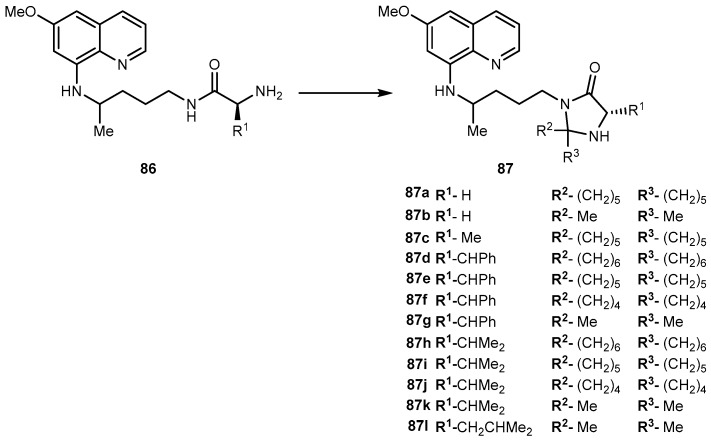
Formation of imidazolidin-4-ones derivatives of **4** (Adapted from [135]).

Some peptide and amino acid derivatives of **4** and other 8-aminoquinoline antimalarials have been synthesized in order to reduce the metabolic oxidative deamination pathway, as well as to reduce the toxicity of parent drug. Despite the encouraging results, it has been shown that amino acid and dipeptide derivatives of **4** are rapidly hydrolyzed to parent drug by aminopeptidases and endopeptidases [37] suggesting that they might undergo extensive hydrolysis to the parent drug in GI tract when is orally administered. With intent to enhance the enzymatic stability of amino acid or peptides derivatives of **4** toward proteolytic degradation, Araújo and co-workers introduced an imidazolidin-4-one formation as a useful prodrug approach to protecting the *N*-terminal amino acid residue. They hypothesized that imidazolidin-4-ones derivatives **87a**–**l** (Figure 36) would release the corresponding amino acid derivative via a non-enzymatic reaction, which in turn, could be enzymatically hydrolyzed to **6** [135].

**Figure 37:**
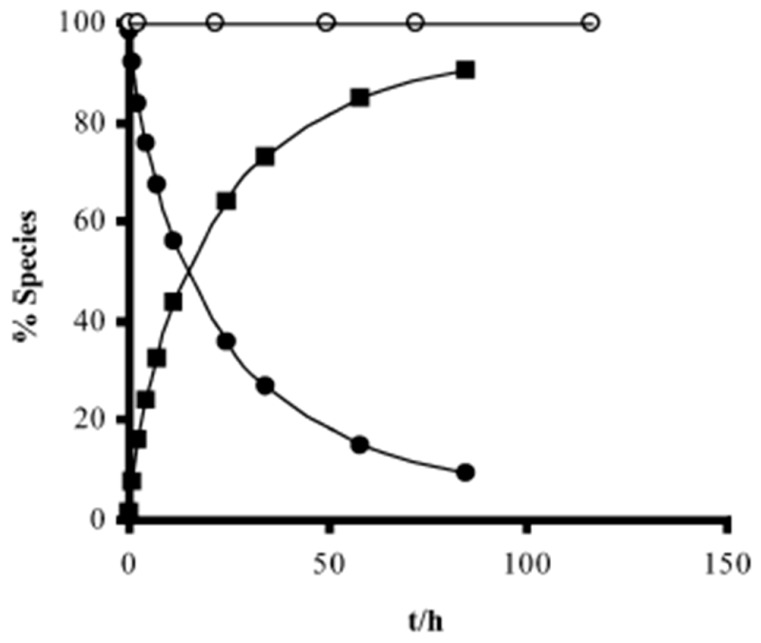
Time courses for components (**87c**) (⚬), Ala-PQ (●) and PQ (■) when (**87c**) and Ala-PQ were incubated in 80% human plasma at 37 °C (reproduced from [135]).

The hydrolysis of imidazolidin-4-one **87a**–**l** in 80% of human plasma was monitored by HPLC for the simultaneous disappearance of substrate and formation of the amino acid derivatives and **6**. With exception of compound **87b**, all imidazolidin-4-ones display unusually high stability when incubated in human plasma, with no significant disappearance of the starting material over 3 days of incubation. Results obtained by Araújo and co-workers showed that stability of **87** is not significantly affected by the R^2^ and R^3^ substituents in the imidazolidin-4-ones moiety. In contrast, the corresponding amino acid derivatives **87**, are hydrolyzed quantitatively to **4** with rates depending on the nature of amino acid chain [135] (Figure 37).

While simple amino acid derivatives behave as prodrugs of **4**, the corresponding imidazolidin-4-ones **87** are too stable to be considered prodrugs. In contrast to their behavior in plasma, the derivatives **87**, hydrolyze to the corresponding amino acid derivatives in pH 7.4 buffer, with half-lives ranging from 9 to 30 days. Moreover, the compounds **87** are hydrolyzed 50–100 times slower than dipeptides or pentapeptides. Araújo et al. thought that the explanation for this observation could be related to the mechanism of hydrolysis [135].

The potential of compounds **87** to prevent the transmission of malaria was studied using a method described above [37]. The parent drug and the imidazolidin-4-one derivatives **87a**–**g** (i.e., those with Gly-, Ala-, and Phe-) completely inhibited the production of oocyst at a dose of 50 μmol/kg. Although imidazolidin-4-one with Val- and Leu-, **87i**–**l**, significantly affecting the sporogonic development of *P. berghei*, it did not completely inhibit the production of oocyst at 50 μmol/kg. At dose of 10 μmol/kg, derivatives **78a**–**c** and **78j**–**l** significantly affects the number of oocysts. This activity was similar to **4** [135].

However, derivatives **87d**–**g** (derived from Phe-) and **97h**, **i** (derived from Val-) did not significantly reduce the oocyst production when compared to control. Araújo and co-workers concluded that the imidazolidin-4-ones derived from Gly-PQ **87a**–**b**, and Ala-PQ **97c** are the most effective gametocytocidal agents, displaying an antimalarial activity reported from glycine derivative of **4** [37], suggesting that incorporation of the imidazolidin-4-one scaffold does not significantly alter s the antimalarial activity. In contrast, imidazolidin-4-one **87** derived from more lipophilic amino acids Phe, Val and Leu was less active when compared to **4** [135]. Vangapandu and co-workers reported that the attachment of a hydrophobic amino acid to the terminal amino group of an analog of **4** led to decreased blood-schizontocidal antimalarial activity, suggesting that the hydrophobic amino acid side chains have a detrimental effect on the activity of 8-aminoquinoline derivatives both in blood schizonts and gametocytes [136]. Imidazolidin-4-ones synthesized are very stable both in chemical and enzymatic conditions, suggesting that they are active per se [135].

**Figure 38:**
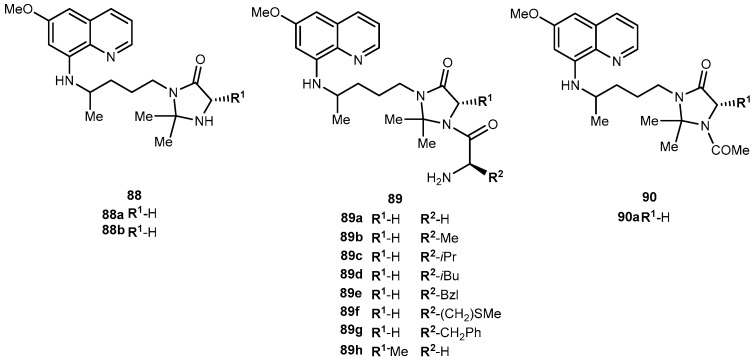
Structures synthesized of imidazolidin-4-one derivative of **4** and tested against *P. berghei*.

In view of the results described above, Vale and co-workers undertook the *N*1-acylation of imidazolidin-4-ones with amino acid in order to (i) fully suppress the hydrolysis of imidazolidin-4-one ring through acylation of its *N*1 nitrogen; (ii) increase the aqueous solubility of novel compounds by insertion of basic amino acid promoieties and; (iii) potentially increase antimalarial activity, given the relevant role usually attributed to the basic amino group of **4**. A set of different structures (Figure 38) was prepared using several amino acid chains (R_1_ and R_2_) in order to check the influence of these substituents on compounds properties.

All compounds **89** were evaluated for in vitro antiplasmodial activity against the chloroquine-resistant *P. falciparum* strain W2. Results demonstrated that: (i) in vivo antiplasmodial activity of compound **89** is not significantly affected by the nature of the amino acid residue at the imidazolidin-4-one *N*1 atom; (ii) activity of compounds **89** is also not significantly affected by the nature of the R1 substituent at the C-5 position of the imidazolidin-4-one moiety **80a** and **b**, had similar values of IC_50_ contrasting with **88a** and **b** in which the incorporation of a methyl group at C-5 leads to complete loss of activity); (iii) acetylation of the imidazolidin-4-one’s N1 atom leads to complete loss of activity (e.g., **90** vs. **89a**), suggesting that the presence of a basic amino group is a major requirement for antiplasmodial activity. In general, compounds **89** displayed greater cytotoxicity than **4** against A549 cells, with exception of **89a** and **89h**, which showed no toxicity. Vale and co-workers believed that the antiplasmodial activity of compounds benefits from the *N*1-acylation of the imidazolidin-4-one ring with an amino acid, which may be partly due to the presence of the primary amino group brought by the inserted amino acid residue. To assess the ability of compounds to inhibit the development of *P. berghei* schizonts in human hepatoma cells (Huh-7 cells), Vale and co-workers used fluorescence activated-cell sorting (FACS)-based method. This method is based on the measurement of the fluorescence of Huh-7 cells, following infection with GFP-expressing *P. berghei* sporozoites. At given time after injection, the percentage of parasitized cells is given by the percentage of GFP-positive events. The extent of intracellular development is proportional to the number of GFP copies in the cell, measured as the intensity of fluorescence. The hepatic anti-plasmodial activity of compounds **89** was monitored by measuring infection of Huh-7 cells incubated with various concentrations of each, 48 h after sporozoite addition. All compounds **89** displayed a marked dose-dependent effect on parasite development. Despite IC_50_ were higher than of 6, several compounds displayed marked antiplasmodial activity. The potential of compounds **89** to inhibit the sporogonic cycle of *Plasmodium* within the mosquito gut was once again studied using a model previously described [37,135]. Compounds were tested at 10 and 50 μmol/kg and although none of them was particularly good at decreasing the percentage of infected mosquitoes, all of them were active at effectively reducing the mean number of oocysts formed per infected mosquito. Moreover, while none of them was superior to **4** at the highest dose, at the lowest dose **89g** and **h** were comparable to the parent drug, and **89a** and **89f** were better than **4**. Compound **89a** significantly reduced the sporogonic development of the parasite at both doses tested, while compound **89b** was inactive at 10 μmol/kg; **89e** was clearly active but did not rank the best of the set, in contrast to its relative activity against liver-stage *P. berghei*. Even though most of the compounds **89** were generally not as active as **4.** Nonetheless, their remarkable chemical and enzymatic stability, as well as preliminary data on ADME properties, suggest that they are promising leads toward novel hydrolytically and enzymatically stable drugs with therapeutic indices superior to those of **4** [137].

**Figure 39:**
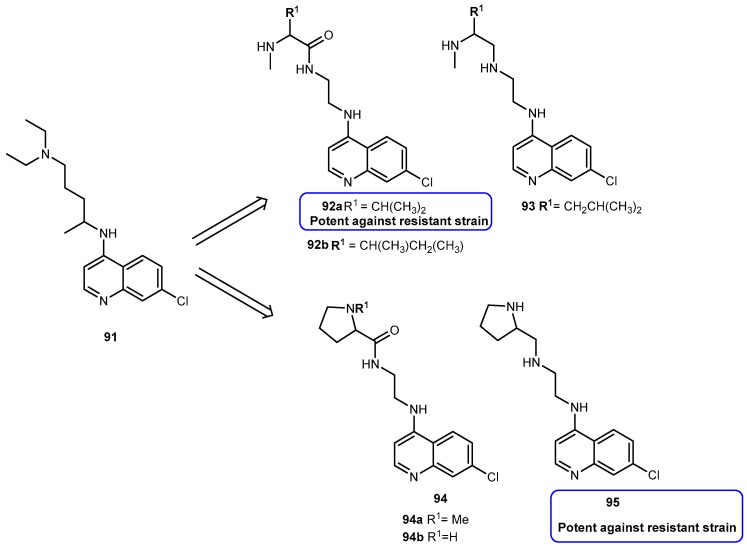
Chloroquine (**91**) and its side-chain modified 4-aminoquinoles (**92**–**95**). Derivative **95** was the most potent against resistant strain wheres **92a** was the most potent against the sensitive strain.

Recently, a novel series of side chains modified 4-amino-quinolines with a reduced amide bond (Figure 39) were developed and its antimalarial activity was assessed on both chloroquine-sensitive (3D7) and chloroquine-resistant (K1) strains of *P. falciparum* [138]. Although chloroquine (**91**, Figure 39) is an important therapeutic agent against malaria, *P. falciparum* strains resistant to **91** has emerged through years in endemic areas [139]. Thus, it is necessary to develop or improve chemotherapeutic agents in order to prevent resistance.

The novel derivatives of **91** designed in order to increase lipophilicity and antimalarial activity. Since amide bond is unstable on biological milieu it was hypothesized that its reduction increased the lipophilicity and therefore might enhance theirs in vivo activity. Yet, so far, the authors did not evaluate the antimalarial activity in vivo. Regarding in vitro activity, the derivatives with a basic nitrogen atom at the side chain showed significant antimalarial activity. The most promising compounds tested, **92a** and **b** showed significant inhibition (IC_50_ 0.28 and 0.31 μM) of parasite growth against K1 of *P. falciparum* whereas compounds **93**, **94b** and **95** (IC_50_ 0.18, 0.22 and 0.17 μM) exhibited a higher activity against this strain as compared to reference drug, **91** (IC_50_ 0.255 μM) [138]. Apparently, the introduction of the methyl group in the side chain (e.g., **94a**) results in increased activity against the 3D7 strain. In fact, this derivative showed better activity than **91** against K1 of *P. falciparum*. The novel derivatives demonstrated less cytotoxicity when incubated in VERO cells and higher selectivity index. It was also demonstrated that all derivatives formed a strong complex with hematin and inhibited the β-hematin formation in vitro*,* suggesting that they act as heme polymerization target [138]. These results suggest that these derivatives might be promising for further lead optimization in order to obtain novel compounds against resistant strains of *P. falciparum*.

#### 3.3.3. Leishmaniasis

Visceral Leishmaniasis (VL) is the most severe form of the disease, being fatal if untreated [139]. Currently, there are no effective vaccines to prevent *Leishmania* infections and, therefore, management of VL relies on chemotherapy with first-line drugs (e.g., pentavalent antimonials) second-line drugs (e.g., pentamidine) or its lipid formulations and miltefosine [140]. However, these drugs have several problems as specific toxicities, elevated costs, prolonged treatment regimens, low patient compliance, and parasite resistance [141]. The antimalarial **4** is known to exhibit activity against visceral leishmania.

**Figure 40:**
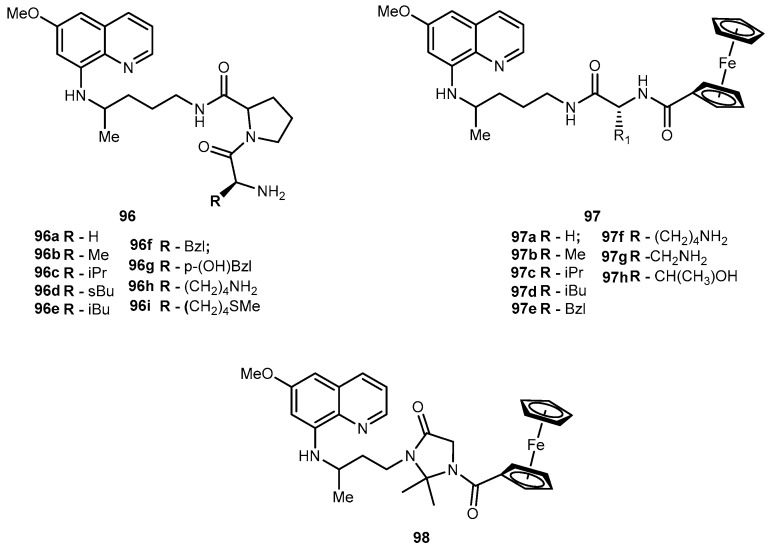
Peptidomimetic derivatives, imidazolidin-4-one derivatives and Ferrocene derivatives of **4** evaluated *for Leishmania infantum*.

In light of previously relevant findings about peptidomimetic derivatives of **87** [135,137], Vale-Costa and co-workers evaluated the compounds previously synthesized as imidazolidin-4-one derivatives **89a**–**c**,**f**–**g**, PQ-Pro-AA derivative (**96**) and ferrocene derivatives of **4** (**97**–**98**, Figure 40), against both the promastigote and intramacrophage amastigote forms of *Leishmania infantum*, the agent of Mediterranean visceral leishmaniasis.

Among the compounds tested, compounds **89c** and **96c** were the most active with IC_50_ similar to **6**. Although these compounds are among the derivatives of **6** with the highest lipophilicity values, there was no direct correlation between clogP values and IC_50_. Compounds **89c** and **96c** are closely related, as compound **96c** is the imidazolidin-4-one peptidomimetic surrogate of dipeptide derivative **84c**, suggesting that valine residue common to the two derivatives is beneficial for in vitro antileishmanial activity against promastigotes [142].

When the compounds were tested for antileishmanial activity inside macrophages using **4**, sitamaquine and miltefosine as reference drugs, compound **96c** was much more active than compound **89c**. This might be related to the fact that compound **89c** is more susceptible than compound **96c** to proteolytic degradation and the fact that protease activity is increased in Leishmania-infected macrophages. Hypothetically, proteolytic degradation would cause the cleavage of the dipeptide moiety in compound **96c**, leading to release of **6**. If this was the case, compound **89c** would be expected to display an activity similar to the parent drug, which did not occur. This fact might be due to macrophage metabolization of compound **89c**, but not **6** or **96c**, to originate a compound which is more toxic to *L. infantum*.

Afterwards, Vale-Costa and co-workers, evaluated the efficacy of compound **87c** in a mouse model of VL which was as effective as **4**, at low doses, at reducing hepatic parasite loads, with no evidence of negative side effects. It was possible observed that peptidomimetic derivatives of **4** were less prone that **6** to metabolic conversion mediated by rat liver enzymes. The peptidomimetic derived **89c** displays higher in vivo antileishmanial activity, lower toxicity, and higher stability compared to its parent drug [142].

Concerning the ferrocenes (Fc) derivatives of **4** (i.e., **97**, **98**), the results obtained for promastigotes were very variable, reflecting the structural diversity of these organometallic compounds. Compounds **97a**–**h**, where **4** is linked to Fc through a variable amino acid spacer, was generally not very promising. One of the most active compounds against *L. infantum* promastigotes was compound **98**, with an activity comparable to that of miltefosine. This is interesting since this compound includes structural modifications imidazolidin-4-one and Fc moiety, which can be seen as an organometallic surrogate of peptidomimetic compound **89**. Similar to peptidomimetics, the most promising organometallic derivatives of **4** against promastigotes were tested in intramacrophagic amastigote form from *Leishmania*. Compound **98** revealed a significant activity in this setting. Nonetheless, the most striking observation was related to the potent activity of **97a** against amastigotes that was not differen from miltefosine. Thus, the presence of an imidazolidin-4-one ring seems to be not necessary for intramacrophagic activity in the case of organometallic derivatives. Most likely the joining of the constrained cyclic imidazolidin-4-one motif with the bicyclic Fc in compound **98**, represents a structure too large or too stiff to easily enter the macrophage and/or the amastigote. By removing the imidazolidin-4-one ring (as in compound **97a**) such problem might be minimized or eliminated, explaining the higher activity of compound **97a** comparatively to **98**. Since these compounds, have interesting or highly potent activities combined with very low toxicity for host cells, they should be considered as leads for the development of a novel and safer antileshmanial derivatives of **4** [142].

### 3.4. Bacterial Diseases

The increasing incidence of infections caused by the rapid onset of bacterial resistance to available antibiotics is a serious health problem. As multidrug-resistant bacterial strains proliferate, the need for effective therapy has stimulated research into the design and synthesis of novel antibacterial/antimicrobial agents [143].

#### 3.4.1. Gram(+) and Gram(−) Microorganisms

Cell permeating antimicrobial agents can potentially play an important role in eliminating infections by intracellular pathogens. Unfortunately, many antibiotic classes do not penetrate the plasma membrane effectively [144].

Ibrahimi and co-workers synthesized amino acid conjugates of fluoroquinolone, ciprofloxacin and norfloxacin, metronidazole and sulfadiazine, and screened for their activity against *Staphylococcus aureus*, *Escherichia coli*, *Pseudomonas aeruginosa* and *Bacillus subtilis*. These strains were used due to their pathophysiological relevance and close relation to pathogenic strains related to human diseases. The antibiotics chosen for chemical modification have a wide range of activity [144]. All agents are considered broad-spectrum antibiotics with activity against Gram(+) and Gram(−) bacteria. However, they present some limitations, for example, fluoroquinolones can cause adverse reactions in central nervous system, skin and gastrointestinal tract [145].

Several conjugation drugs showed greater inhibitory activity compared to the parent drug, which may be a result of several independent or combined mechanisms. Probably, the conjugate may increase the concentration of the compound inside the cell and block the compound sites that interact with antibacterial resistance proteins, thus preventing the inactivation of the drug. Lipophilicity is higher for conjugates comparatively to the corresponding parent antibiotic, which is important since it is believed that the strong lipophilic character of a drug plays an essential role in producing an antimicrobial effect, being related to membrane permeation in biological systems [144].

**Figure 41:**
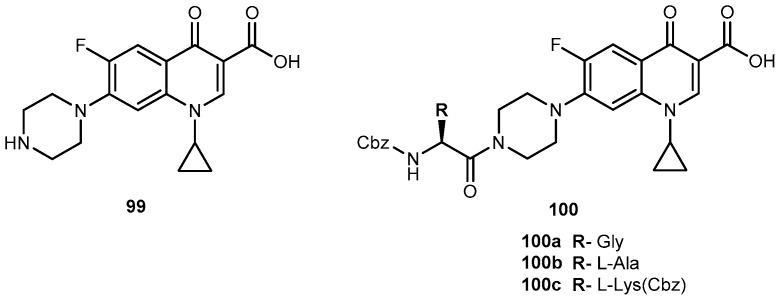
Structures of ciprofloxacin (**99**) and amino acid ciprofloxacin (**100a**–**c)** conjugates.

Ciprofloxacin (**99**) and its conjugates (**100a**–**c**) Figure 41) were screened and showed growth inhibition in all strains. At lower concentrations, **99** demonstrated to be highly active and might be considered a much more potent antibiotic. Despite the good activities, this conjugate family showed a variable result among strains, where **100a** and **100b** inhibited over 90% of growth in *B. subtilis* and **100b** did not show significant inhibition in any strain and **100a** only inhibits 50.26% of growth in *S. aureus* [144].

**Figure 42:**
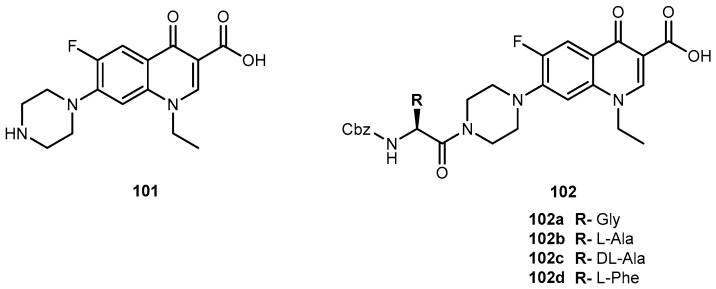
Structures of norfloxacin (**101**) and amino acid norfloxacin conjugates (**102a**–**c**).

Norfloxacin (**101**, Figure 42) inhibited the growth of all screened strains but the strength of its conjugates is variable among bacteria strains. At 60 μg/mL, conjugates **102a**–**d** inhibited a higher percentage of *S. aureus* growth than the parent antibiotic whereas in *P. aeruginosa*, only **102a** shows activity [144].

**Figure 43:**
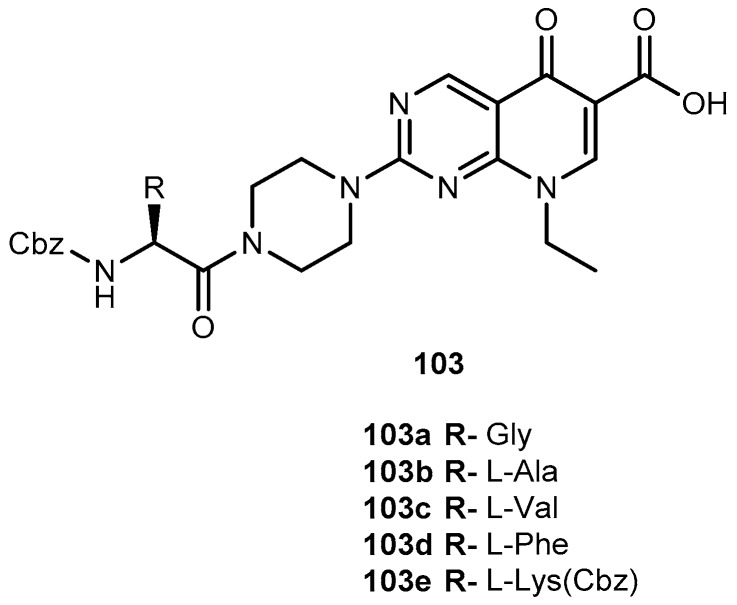
Structures amino acid pipemidic conjugates (**103a**–**e**).

The pipemidic acid derivatives of **102** (Figure 43) were active only against Gram(+) bacteria as *S. aureus* and *B. subtilis*. *B. subtilis* showed high sensitivity to all pipemidic conjugates at a concentration 40 μg/mL. Prodrugs **103a** and **103e** also presented higher growth inhibition than the parent antibiotic. At a fixed dose 200 μg/mL, *S. aureus* showed the greatest sensitivity to conjugates **103c** and **103d** [144].

**Figure 44:**
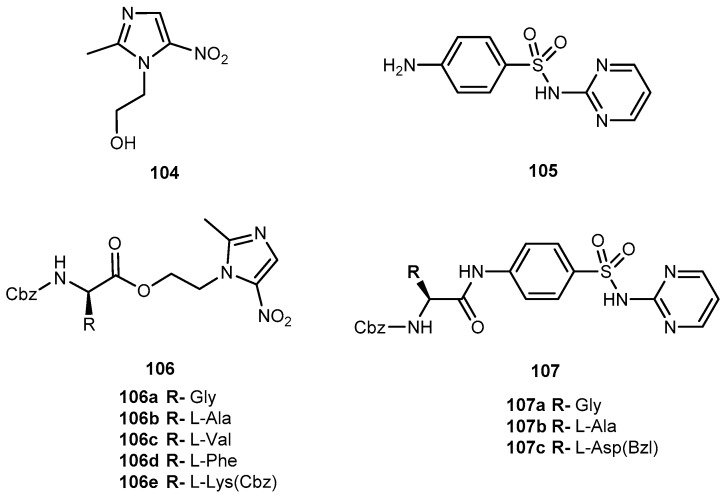
Structures of metronidazole (**104**) and its conjugates (**106a**–**e)** and sulfadiazine (**105**) and its conjugates (**107a**–**c**).

Finally, metronidazole (**104**), sulfadiazine (**105**) and conjugates (**106**–**107**, Figure 44) did not inhibit the growth of any strain [144].

Ibrahimi and co-workers considered that the inclusion of amino acids in antibiotics, may provide alternative options for treatment of bacterial-resistant strains, with the benefit of increasing drug uptake and/or eliminate or alleviate adverse side effects [144].

#### 3.4.2. Tuberculosis (TB)

The emergence of drug-resistant tuberculosis worldwide is alarming [146]. It is estimated that one-third of the world’s population is currently infected with *Mycobacterium tuberculosis* (*M. tuberculosis*), and every year 8–9 million new cases arise. In addition, almost 500,000 people per year are infected with multi-resistant TB (MDR-TB) and 40,000 new cases of drug-resistant TB (XDR-TB) are estimated. XDR-TB was defined as a resistance to any fluoroquinolones and to at least one of the three injectable drugs capreomycin, kanamycin or amikacin. Moreover, unlike MDR-TB, XDR-TB is often untreatable. In addition, HIV co-infection with MDR-TB in immunocompromised patients is a serious challenge. Therefore, it is urgent develop novel types of drugs or prodrugs with a new mechanism of action [147].

**Figure 45:**
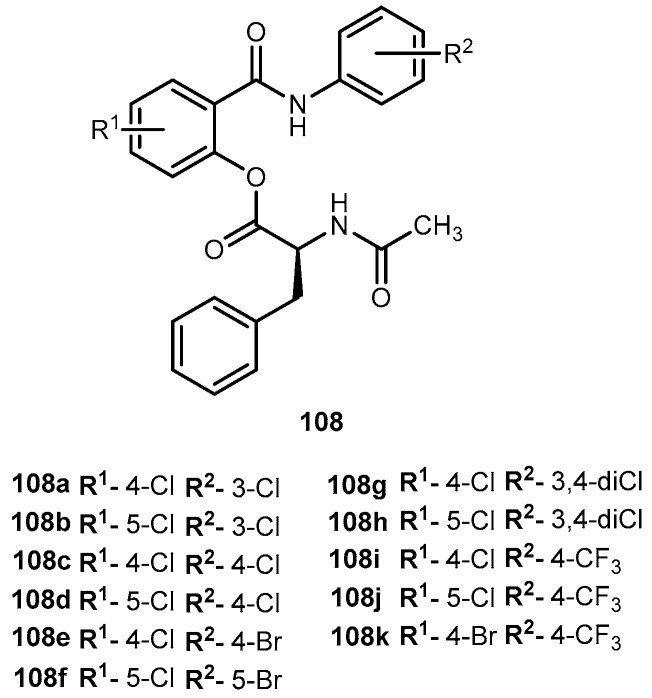
Structures of halogenated salicylanilides and *N*-acetyl-l-phenylalanine tested against *M.* *tuberculosis* strains [148].

A novel series of halogenated salicylanilides and *N*-acetyl-*L*-phenylalanine (**108a**–**k**, Figure 45) were synthesized and their antibacterial activity was assessed against *M. tuberculosis* including MDR-TB strains and atypical mycobacteria [148].

The novel esters showed high antituberculous activity (minimum inhibitory concentration, MIC 0.25 μmol/L). Generally, the compounds synthesized were more active than the starting salicylanilides, with exception for **108j** that was less active. The derivatives **108c** and **108d**, that present similar MIC values, were more active on *M. tuberculosis* than salicylanilides, in some cases MIC decrease 8-fold (e.g., **108e**, **g** and **92i**) [148]. As mentioned, it seems that lipophilicity influences the activity, but the relationship is not direct. In general, the most lipophilic **108g**, **i**, **k** and partly **e** showed the highest anti-mycobacterial activity [148].

The MDR-TB results (MIC 1–2 μM) of **108 g**, **k** and **i** suggesting that they might be promising candidates for MDR-TB treatment. Comparing **108i** and **k**, the late had almost the same antitubercular efficacy as **108i**. Based on these results, Krátý and co-workers considered **108k** an optimal candidate for further investigation as a promising antimycobacterial agent. In general, to increase the activity against bacteria it seems that the most convenient substituents are 4-CF_3_ (e.g., **108i** and **k)** and 4-Br (e.g., **108f**). Through comparison of substituents on the salicylic ring, those that present 5-Cl rather 4-Cl are more active against bacteria, with exception of **108i**. Therefore, some of these esters could be potential agents against Gram-positive infections [148]. Considering the results, salicylanilides of *N*-acetylphenylalanine esters could be a new highly active group with promising wide-range antibacterial activity [148].

#### 3.4.3. Leprosy

**Figure 46:**
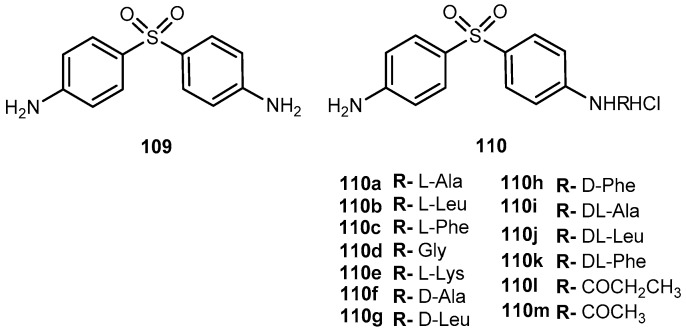
Structures of dapsone (**109**) and its amino acid amines derivatives (**110a**–**m**).

Dapsone (diamino-diphenyl sulfone) (**109**, Figure 46) is a bacteriostatic and antileprosy agent with a low aqueous solubility. In order to overcome this limitation, Pochopin and co-workers developed novel amino acid amides derivatives of dapsone **110a**–**m** (Figure 46).

Through chemical stabilities studies of prodrugs, the only degradation product observed at any pH value was the parent drug, suggesting that no other routes of degradation apart from hydrolysis of the amide bond occur [149]. The addition of the amino acid residues to **109** resulted in improvement of 2–3 orders of magnitude in water solubility for the HCl salts, with exception of *L*-Phe-dapsone **110c**. *L*-Lys-dapsone **110e**.The greatest solubility over the widest pH range might be due to the ionizable α-amino which is protonated at physiological pH. The derivatives **110a**–**e** were all substrates for leucine aminopeptidase. The corresponding *D*-amino acid derivatives were completely stable in the presence of leucine aminopeptidases over 8 h, which is consistent with the specificity of leucine aminopeptidases for amino acids in *L*-configuration, excluding glycine, which does not have a chiral center. Hydrolysis of **110a**–**e** to **109** occurred in human plasma and human, rat, and rabbit blood. Of all compounds studied, derivative **110e** presented the best results concerning to half-life (>2 years at pH 4) and is rapidly and quantitatively hydrolyzed in the systemic circulation after intravenous administration. Therefore, **110e** might a good prodrug example for further development of other poorly soluble amides [149].

### 3.5. Brain and Central Nervous System (CNS) Diseases

Currently, one of the most significant challenges in pharmaceutical research is the development of new drugs targeting the central nervous system (CNS). Despite recent advances in brain research, adequate efficient drug therapies against many CNS disorders have not been discovered. A successful treatment of the CNS diseases requires not only the identification of appropriate targets within the CNS but also strategies to improve the transport of therapeutics across the relatively insurmountable blood brain barrier (BBB) into the CNS at sufficient levels to achieve the desired effect. It has been estimated that more than 2% of the small molecular weight drugs and practically none of the large molecules weight drugs developed for CNS disorders cross the BBB at adequate levels [150]. The carriers for glucose (glucose transporter 1,GLUT1) and large amino acid (LAT1), have been found to have a sufficiently high transport capacity to hold promise for significant drug delivery to the brain [151,152].

#### 3.5.1. Epilepsy and Bipolar Disease (Anticonvulsants)

Epilespsy is not one condition, but a complex set of cerebral disorders that in common the occurrence and recurrence of seizures [153]. Nowadays, about 65 million people worldwide live with epilepsy, and only 70% of them control the seizures with the available medication [154], at expenses of the significant adverse side effects that increase their toxic actions when a lifelong medication is required [155]. The remaining 30% of patients are still resistant to anticonvulsant drugs [156].

**Figure 47:**
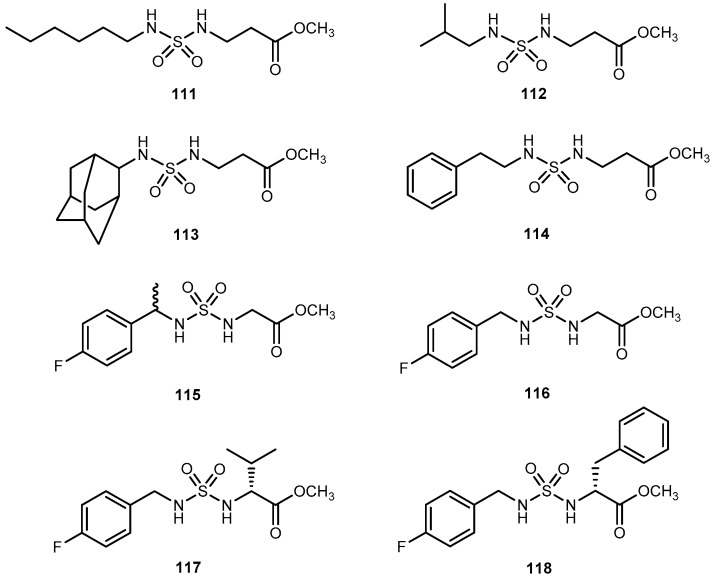
Amino acid derived sulfamides evaluated on maximal electroshock seizure.

Recently, a novel series of β-alanine derived sulfamides (**111**–**116**), and derivatives that had *L*-valine and *L*-phenyl alanine skeleton (**117**,**118**) instead β-alanine moiety of methyl [*N*-(*p*-fluorobenzyl)-sulfamoyl]-β-alaninate were synthesized and assessed for maximal electroshock seizures (MES) (Figure 47).

Despite all the derivatives showed protection against the maximal electroshock seizure test in mice at the lowest dose tested (30 mg/kg). They did not show significant protection against chemical-induced convulsion by pentylenetretrazole (PTZ) [157]. It should be pointed out that most of the structures did not induce toxic effects, which support the use of amino acids for design and development of novel anticonvulsant compounds. Based on biological results, it seems that the substitution of the sulfamide moiety by branched alkyl groups lead to increase of anti-MES activity [157].

**Figure 48:**
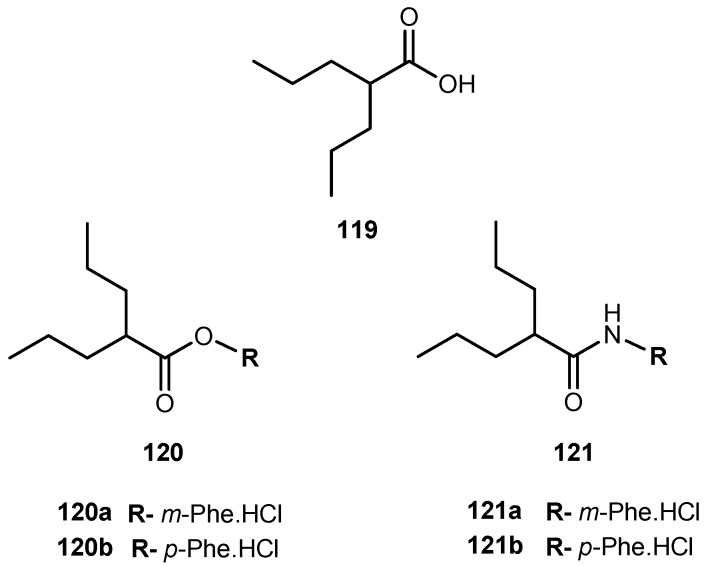
*L*-Phenylalanine amide (**121a**–**b)** and ester prodrugs (**120a**–**b)** of valproic acid (**119**)**.**

Peura and co-workers synthesized and evaluated novel ester *L*-phenylalanine amide and ester prodrugs (**120a**–**b** and **121a**–**b**) of valproic acid (**119**, Figure 48), an anticonvulsant used in the treatment of epilepsy and bipolar mania which is extensively bound to plasma proteins, metabolize to toxic species in the liver and is assymmetrically transported across the BBB [158,159,160]. The main aim of Peura and co-workers was to demonstrate the potential of the amino acid as carriers for the drug targeting the CNS via LAT1 [161].

The stability of the prodrugs synthesized was evaluated in aqueous buffer (pH 7.4) and their bioconversion to the parent drug was determined in rat brain (20%), rat liver homogenates (50%) and human plasma (75%). All prodrugs demonstrated adequate chemical stability. Ester derivatives **120a**–**b** were rapidly hydrolyzed to parent drug in tissue homogenates and plasma. On the other hand, the enzymatic hydrolysis of amides **121a**–**b** in brain, liver homogenates and plasma resulted only in a slight degradation of the prodrugs in vivo. Although the biodegradation of **121a**–**b** was determined to be quite slow, Peura and co-workers thought that was reasonable to determine their transport-binding mechanism and the brain uptake. In fact, the slow release of an active drug in CNS might provide a sustained therapeutic effect for a prodrug [161].

The transport mechanisms across the BBB and the LAT1 affinity of the prodrugs **120a**–**b** and **121a**–**b** as well the amount of the prodrug uptake into the brain were determined by using the modified in situ rat brain perfusion technique. All prodrugs were able to bind the LAT1 at the BBB. The regioselective positioning of valproate in the *L*-phenylalanine structure significantly affects the binding of the prodrug to LAT1. There were no significant differences between the affinities to LAT1 whether **119** was linked to the promoiety by amide or ester bond. However, a significant difference between affinities of the prodrug to LAT1 was observed when **119** was linked to the para- or meta-position of *L*-phenylalanine promoiety. The affinities of **120a** and **121a** were more than 10-fold compared with those of **120b** and **121b** [161].

Reversibility of the prodrugs by rat LAT1 was also examined to confirm that prodrugs do not inhibit the LAT1 transport irreversibly since this parameter is an essential requirement for the brain uptake of prodrugs via specific transporters. Thus, total amounts of all the prodrugs in the brain after the in situ rat brain perfusion were studied to confirm that all prodrugs do not only bind to LAT1 but are also transported across BBB into the brain. The valproate prodrugs were able to cross the rat BBB and gain entry into the brain [161].

Amide prodrugs **120b** and **121b** were shown to cross the rat BBB more efficiently than the esters **120a** and **121a**. These difference between these two groups of compounds may be due to the exposure of the esters to the degrading enzymes at the BBB during the perfusion. Nonetheless, the difference between the rate of uptake of **120b** and **121b** is not significant and could be explained according to their affinities. One plausible explanation for the limited uptake capacity of prodrug **120a** may be related to steric restriction. In addition, this prodrug may strongly bind to LAT1 that it does not leave the transporter relatively as easily as prodrug **121b**. Thus, *L*-phenylalanine derivatives, especially substituted in meta-position of the phenolic group could be highly used as prodrug designed for LAT1-mediated CNS delivery of small molecular weight drugs with poor brain penetration properties [161].

**Figure 49:**
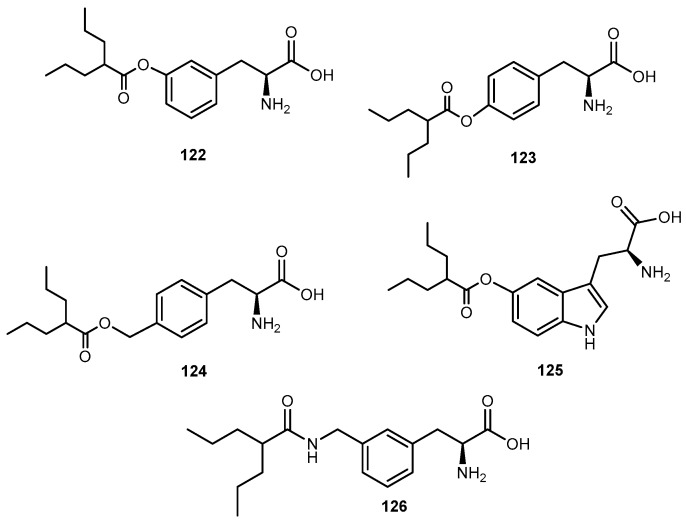
Chemical structures of amino acid esters (**122**–**125**) and amide prodrug (**126**) of **119** studied by Gynther et al. [161].

More recently, seven amino acids prodrugs of **119** targeted for LAT1 were evaluated in order to provide insights related to brain uptake and systemic pharmacokinetics of parent drug. In general, all prodrugs presented affinity for LAT1, with prodrugs that were derivatized at the *meta*-position of phenylalanine moiety exerting higher inhibitions than prodrugs that were derivatized at the *para*-position. Their bioconversion in vitro revealed that ester prodrugs (**122**–**125**, Figure 49) were converted to parent drug in rat and human-derived biological media more effectively than their corresponding amide prodrugs **121a**, **121b** and **126** (Figure 49).

Since esters prodrugs were too unstable to be studied in a rat model, only the corresponding uptake of amides was assessed in vivo. The amide prodrugs bound to human plasma proteins to a greater than **119** and this linkage is independent of prodrug concentration. It was also demonstrated that amide prodrugs were delivered into the brain after intravenous bolus injection of equivalent doses. One of amide prodrugs, **126**, achieved a great brain uptake and high selectivity for LAT-1 and released parent drug slowly into the brain. Therefore, this prodrug has a great potential to stabilize the parent drug concentration within the brain [162].

**Figure 50:**
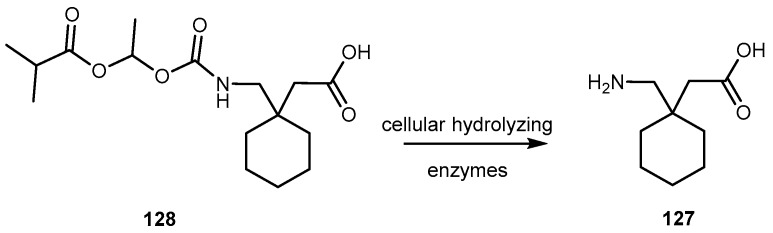
Bioconversion of XP13512 (**128**) to parent drug (**127**) by cellular hydrolyzing enzymes (Adapted from [4]).

Gabapentin (**127**, Figure 50) is a structural analog of GABA (gamma amino butyric acid) used for the treatment of epilepsy [163] and postherpetic neuralgia [164]. The drug has structural features of amino acids and has also shown efficacy in the treatment of anxiety disorders [165] and diabetic neuropathy [166]. The bioavailability of **127** is dose-dependent, decreasing from approximately 60% (at a 300 mg dose) to 35% or less (at doses are used to treat neuropathic pain). The underlying mechanism of this dose dependence is thought to be saturation of absorption of **127** that occurs in the intestine [167]. The absorption of **127** in the upper small intestine is mediated by the low-capacity solute transporter, possibly an L-type amino acid transporter. Following oral absorption, the drug is rapidly excreted in urine and therefore, **127** must be administered three or four times per day to maintain therapeutic levels [168]. Efforts to develop a sustained release formulation for **128** have failed, primarily due to the lack of significant absorption of the drug in the large intestine [169].

The prodrug of **127** was stable at physiological pH but was rapidly converted to the parent drug in intestinal and liver tissues from rats, dogs, monkeys, and humans. In addition, **128** was not a substrate or inhibitor of major cytochrome P450 isoforms. The prodrug had an active apical to basolateral transport across Caco-2 cell monolayers and pH-dependent passive permeability across artificial membranes. In addition, it inhibited the uptake ^14^C-lactate by human embryonic kidney cells expressing monocarboxylate transporter type 1 (MCT-1) as well as inhibited uptake of ^3^H-biotin into Chinese Hamster Ovary cells overexpressing human sodium-dependent multivitamin transporter (SMV1), suggesting that transport of prodrug is mediated through these transporters [170].

**Figure 51:**
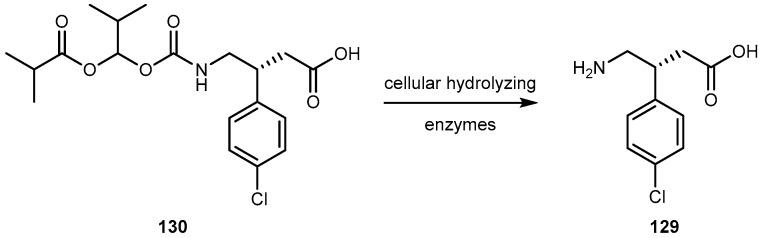
Bioconversion of XP19986 (**130**) to *R*-baclofen (**129**) by cellular hydrolyzing enzymes (adapted from [4]).

Baclofen (**129**, Figure 51) is a racemic GABA_B_ receptor, used for treating spasticity and other neurological diseases. Similar to **127**, **129** has structural features of amino acid. In humans, **129** is rapidly absorbed after oral administration and the drug is rapidly eliminated, largely unchanged, via renal excretion. Moreover, it is only absorbed in the upper small intestinal by the saturable active transport mechanism. Similarly to **128**, **129** needs to be administered frequently to maintain its therapeutic effects [171]. In order to overcome these limitations, Lal et al. developed a new prodrug of **129**, XP19986 (**130**, Figure 51), which is a pharmacologically active *R*-isomer of **129**. Unlike the parent drug, this prodrug was designed to be well absorbed from the colon, allowing the drug to be delivered in a sustained release formulation that may reduce the frequency of administration and reduce fluctuations in plasma exposure. This, in turn, may lead to improved efficacy through a combination of a greater duration of action, greater subject convenience and therefore, adherence, and improved safety profile compared with **129** [172].

The prodrugs **128** and **130** (of **127** and **129**, respectively) further exemplify the utility of amino acid prodrugs targeted to transporters other than oligopeptide transporters expressed in the gastrointestinal tract [4].

**Figure 52:**
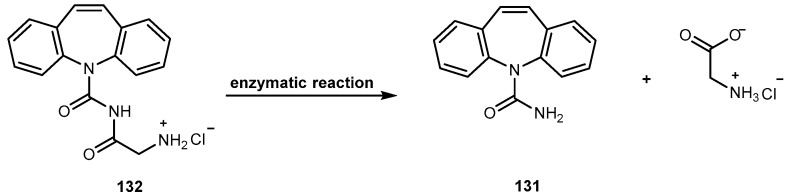
Bioconversion of *N*-Gly-CBZ (**132**) to CBZ (**131**) in vivo (Adapted from [4]).

The anticonvulsant carbamazepine (**131**, Figure 52) is the most frequently prescribed first-line drug for the treatment of partial and generalized tonic-clonic epileptic seizures [173]. It is effective as a treatment of epilepsy in the majority of cases, but it hampers the parenteral administration of the drug [174]. It has been reported that **131** has slow and somewhat erratic oral absorption and presents incomplete oral bioavailability in some cases. Hemenway and co-workers synthesized a novel water-soluble analog of **131**, *N*-Gly-CBZ (**132**, Figure 52) where the amino acid glycine has been linked to the urea nitrogen of parent drug, leading to a peptide link acyl urea functionality. The stability of **132** was found to range over four orders of magnitude with the greatest stability in the pH range 3–4 where an injectable solution of >50 mg/mL of **131** equivalent (697 mg/mL) appeared to be possible. The introduction of *N*-Gly increased the aqueous solubility of the parent drug. However, due to its chemical instability (t_90%_ = 5.9 days at pH 4.0, 25 °C), the parenteral formulation of prodrug was limited only to the freeze-dried product for reconstitution prior to use [175]. The enzymatic stability in vivo performance of **132** in rats after oral administration was evaluated by Hemenway and Stella [176].

The stability of the prodrug in rat blood and plasma was adequate to ensure minimal *ex-vivo* conversion during blood sample processing and analysis, allowing pharmacokinetic studies to be conducted in rats. The prodrug was readily converted to the parent drug following IV administration to rats (Figure 51).

The rate of appearance of **131** following IV administration of the prodrug was approximated by a t_1/2_ value of about 1 min, which is very similar to the t_1/2_ value for prodrug elimination. The prodrug gives an equivalent dose of the parent drug compared to an equimolar dose of **131** as control following IV dosing. This result is consistent with **132** acting as a viable prodrug candidate with rapid and complete conversion to the parent drug using the rat animal model. The oral administration of **132** resulted in the appearance of plasma levels of **131** but not prodrug in rat blood samples. It appears that most of the conversion occurs prior to **132** reaching systemic circulation. It is likely that a high degree of **132** bioconversion to **131** occurs in GI tract, facilitated by non-specific peptidases present in high levels in the lumen and at the brush border of intestinal mucosa. The prodrug also demonstrated superior oral bioavailability due to its greater aqueous solubility [176].

**Figure 53:**
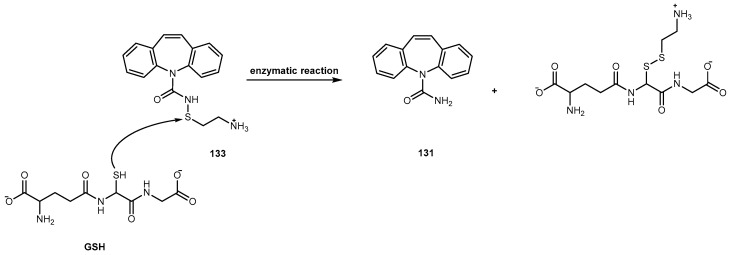
Possible conversion mechanism of *N*-cysteamine carbamazepine **(133)** to the parent drug **131** by the nucleophilic attack by GSH (adapted from [4]).

Another prodrug of **131**, *N*-cysteamine carbamazepine (**133**, Figure 53), has also been developed for IV use. The aqueous solubility of the *N*-cysteamine derivative exceeded 100 mg/mL, at pH 2.6, and its predicted shelf-life (t_90%_) was 320 days, at pH 4.0 and 25 °C. After an IV dose of **133** in rats, the parent drug was immediately seen in plasma, whereas no intact drug was observed, suggesting that the conversion of prodrug to parent drug occurred rapidly and most probably via reaction with glutathione (Figure 53) [177].

#### 3.5.2. Anxiety Disorders

**Figure 54:**
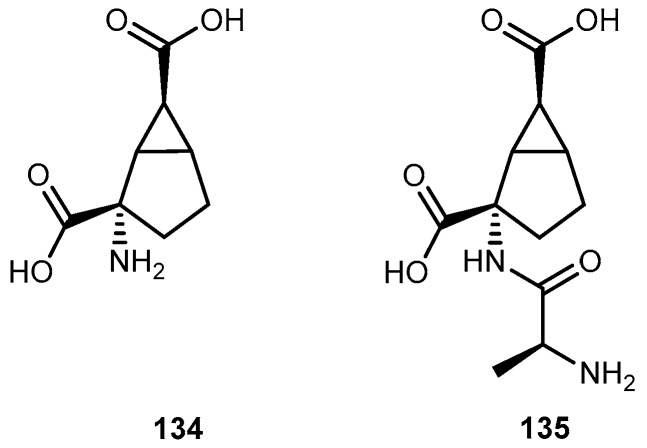
Chemical structures of LY354740 (**134**) and its amino acid prodrug LY544344 (**135**).

LY354740 or eglumetad (**134**, Figure 54) was an investigational drug, which was expected to be useful in the treatment of many psychiatric diseases, including psychosis and anxiety [178]. However, due to its low membrane permeability, **134** presents limited oral bioavailability. In order to overcome this limitation, an amino acid prodrug was developed, LY544344 (**135**, Figure 54), by introducing an alanyl promoiety attached to the amine of the parent drug.

After oral administration of **135** there was a 10-fold increase in brain, plasma and cerebrospinal fluid (CBS) concentrations. The rate of increase in **134** concentration was faster in dose group of **135**, consistent with rapid intestinal absorption of the prodrug via active PepT1-mediated transport. Initial uptake of parent drug into the brain was similarly rapid, but there was a large period of at least several hours between the peak concentrations in plasma and brain. Despite the rapid appearance of parent drug in the CNS, the concentrations were relatively low in brain and CSF compared with plasma, indicating limited passive penetration of **134**. Furthermore, Rorick-Kenh and co-workers compared the oral efficacy of the **134** and its prodrug in rodent models of psychosis and anxiety. Orally administered prodrug (30 mg/kg) and subcutaneously administered parent drug (10 mg/kg) were able to attenuate stress-induced hyperthermia in DBA/2 mice, with the prodrug producing anxiolytic effects at lower oral doses than the parent drug. Oral administration of parent drug did not significantly affect fear-induced suppression of operant responding in rats. However, subcutaneously administration of **135** and orally administered prodrug produced significant anxiolytic effects in this model [179].

Varma and co-workers attempted to elucidate the mechanism of intestinal absorption of **134** and its prodrug **135**. Transport of both compounds was affected by P-glycoprotein-mediated efflux, as shown in transport and uptake inhibition studies in MDCK multidrug resistance 1-transfected cell line and inverted membrane vesicles. Their results suggest that prodrug is (i) a substrate for the intestinal apical uptake PepT1 transporter and for enterocytic peptidase(s) that cleave the peptide bond to release parent drug; (ii) has an affinity for specific efflux transporter present on the apical membrane; and (iii) is able to use the paracellular pathway. The **134** has limited entry into enterocytes and its transepithelial transport is via the paracellular pathway [180]. Despite these results, the clinical development of **135** was recently discontinued [4].

#### 3.5.3. Attention Deficit/Hyperactivity Disorder (ADHD)

**Figure 55:**
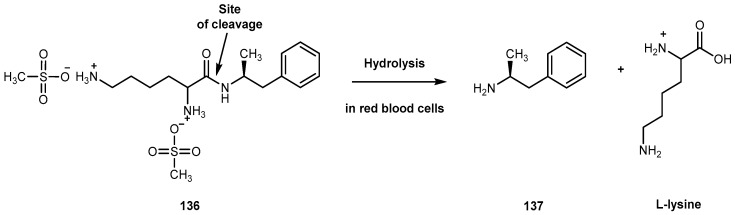
The enzymatic conversation of LDX (**136**) to active *D*-amphetamine (**137**) (adapted from [182]).

Lisdexamfetamine dimesylate (LDX, Vyvane^®^, **136**, Figure 55) is an *L*-lysine amino acid amide prodrug which is used as a psychostimulant for attention-deficit/hyperactivity disorder, a neuropsychiatric condition, in children aged 6–12 years and adults [181]. Unlike other formulations that rely on mechanical release of the active drug that may be affected by GI factors, such as transit time and pH, **136** is a prodrug that has a biological mechanism of drug delivery that uses enzymatic hydrolysis to convert the therapeutically inactive molecule to the active drug, *D*-amphetamine (**137**, Figure 55). The naturally occurring amino acid *L*-lysine is a by-product of the hydrolysis [182].

The prodrug has been shown to provide a long duration effect allowing a once-daily dosing [183] and is the only prodrug available for treatment of ADHD. Therefore, it is important to understand the absorption and enzymatic conversion of intact **136** to the active moiety, **137**. With this in mind, Pennick conducted studies in order to investigate the absorption and enzymatic conversion of **136** in rat and human tissues and cell culture models [182].

Pharmacokinetic studies in portal and jugular vein-cannulated rats showed that after administration of a single dose of **136**, intact prodrug was rapidly absorbed from GI tract. **137** was measurable in both portal and systemic plasma shortly after dosing, suggesting a rapid conversion of **136** to **137** in both portal and systemic circulations, peaked between one and two hours post dosing. The post blood levels of **136** were approximately 10-fold higher than systemic levels, indicating the occurrence of pre-systemic conversion of **136** to **137** in a rat model. The absorption of **136** likely occurs via high-capacity carrier-mediated transport system involving PepT1 in the small intestine. The subsequent metabolism of the drug to its active metabolite by red blood cells also appears to be via the high-capacity system. Thus, saturation of enzymatic conversion of **136** in the blood is unlikely to occur at therapeutic doses. The findings of high-capacity absorption and enzymatic conversion may contribute to the consistent and reproducible pharmacokinetic profile of **136** in humans. The biotransformation process, and not the dissolution of intact **136**, appears to control the rate of delivery of active **137** [182].

#### 3.5.4. Parkinson’s Disease (PD)

**Figure 56:**
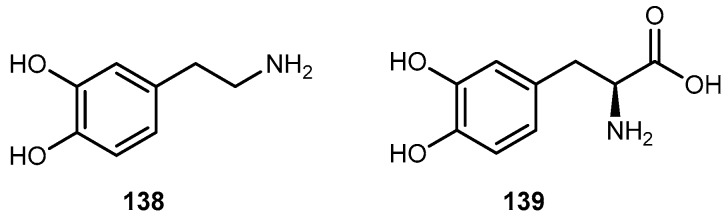
Chemical structure of dopamine (**138**) and its prodrug levodopa (**139**).

Parkinson’s Disease is a degenerative disorder of the CNS that presents with resting tremor, bradykinesia, postural instability, and rigidity and is associated with the loss of dopamine (**138**, Figure 56) in the nigro-statal dopamine system [184]. Prodrugs of **138** were developed since several pharmacokinetic problems were found in its use to treat Parkinson’s disease. First, both amino-terminal and the catechol hydroxyl groups of **138** are rapidly and extensively metabolized after oral administration. Furthermore, **138** is largely ionized, and thus, it is not able to cross the BBB via passive diffusion [185].

Levodopa (**139**, Figure 56) is the only clinically used prodrug of **138** that is transported into the brain via LAT1-transporters as a “pseudonutrient”. The “pseudonutrient” structure of **139** is metabolically transformed to the parent drug in the brain, while its metabolic transformation in peripheral tissues is prevented by simultaneous administration of peripheral inhibitors of **139** metabolizing enzymes, i.e., aromatic *L*-amino acid decarboxylase (AADC) and catechol-*O*-methyltransferase (COMT). The need for these inhibitors complicates treatment with **139**. Furthermore, chronic treatment with **139** has many problematic pharmacokinetic obstacles, e.g., extensive metabolism, plasma fluctuations, erratic oral absorption and variability in the extent of the first-pass effect. In advanced Parkinson’s disease, an extensive loss of nigrostriatal dopaminergic neurons leads to a significant decreased of AADC activity that is essential to convert **139** to **138**. However, **139** with AADC- and COMT inhibitor still remains the cornerstone of the drug treatment of Parkinson’s disease [185].

**Figure 57:**
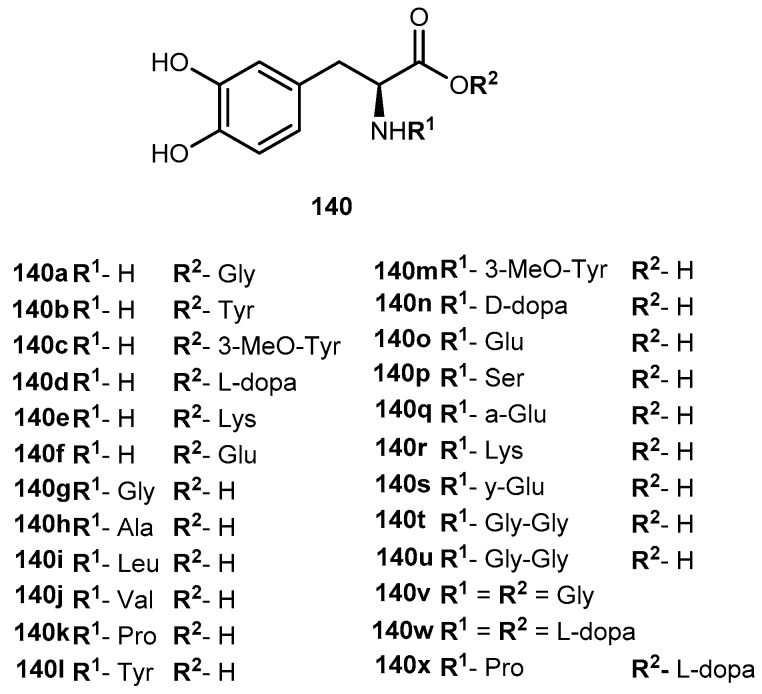
Di- and tripeptides prodrugs of **140**.

Despite the improvement of symptoms during therapy with **139** in Parkinson’s disease patients, the drug has several disadvantages, as mentioned above. This has prompted the search for compounds that could replace **139** in the treatment of this neurodegenerative disease. **139** offers several possibilities for derivatives formation containing biodegradable linkages and useful for prodrug approach. Felix et al. proposed and examined antiparkinson activity in mice of a series of di- and tripeptides (**140a**–**x**, Figure 57) containing **139** [186].

Some of the peptides were more effective in reversing reserpine-induce catatonia and were relatively non-toxic and resulted in a low degree of stereotypic behavior than **140** [186].

**Figure 58:**
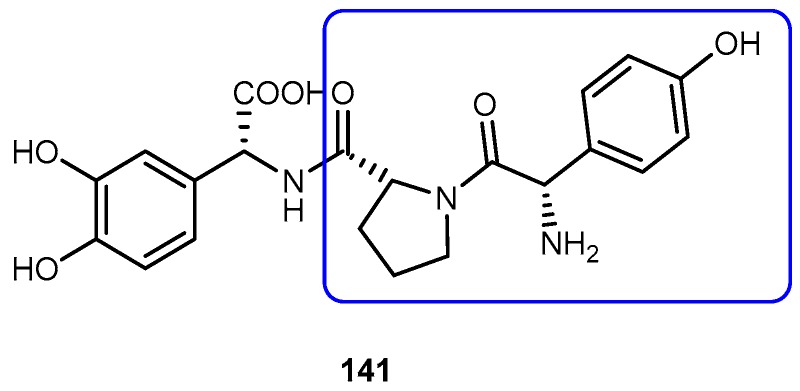
Tripeptide mimetic prodrug of **138**. A d-*p*-hydroxyphenylglycine-l-proline group (blue square) was attached to parent drug.

Wang and co-workers developed a tripeptide mimetic prodrug of **138** (**141**, Figure 58) as a delivery system for improving oral absorption, in which d-*p*-hydroxyphenylglycine-l-proline was attached to **140** [187].

**Figure 59:**
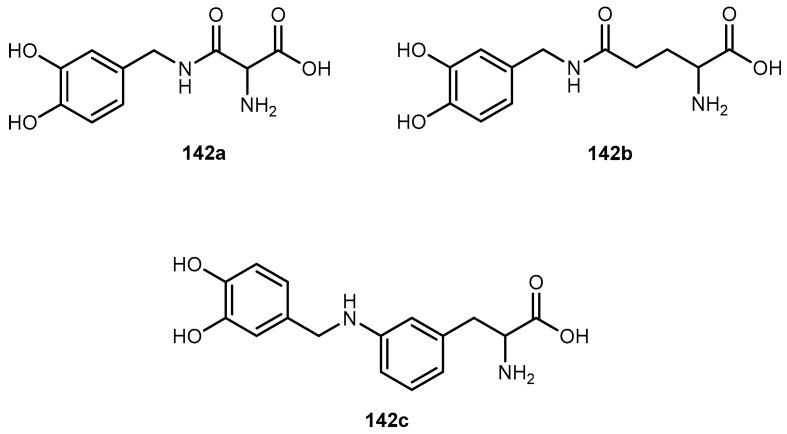
Amino acid prodrugs of **142** synthesized by Peura and co-workers [188].

The tripeptide was well absorbed during a perfusion study in rat intestine, devoid of side effect of **138** on isolated muscles and significantly decreased the methamphetamine-induced rotational behavior in nigrostriatal-lesioned rats, suggesting that it might be an effective agent for Parkinson’s disease [187]. In order to enhance the brain uptake of cationic dopamine, Peura et al. synthesized three amino acid prodrugs of **138** (**142a**–**c**, Figure 59) and evaluated their physicochemical properties, as well as (i) their LAT1-binding and BBB permeation; (ii) brain uptake after IV administration and releasing of **138** ability in rat brain after intraperitoneal administration [188].

Synthesized dopamine prodrugs **142a**–**c** possess affinity for LAT1 at rat BBB during the in situ rat brain perfusion and evoked a sustained release of the parent drug as shown by in vitro stability studies fulfilling the specifications required for tissue-targeted prodrugs. The prodrugs appeared to be reasonably stable in the aqueous buffer at pH 7.4 having half-lives of approximately 10 h, in case of prodrug **142b** or longer, **142a** and **142c**. The half-lives of prodrugs in rat plasma and liver homogenates were notably shorter than those determined in the aqueous buffer solution, suggesting that the prodrugs released the parent drug predominantly enzymatically. Peura and co-workers showed that only the meta-substituted phenylalanine promoiety attached to **138** via an amide bond as **142c** exhibited adequate affinity for LAT1 for carrier-mediated brain uptake. The aspartic acid of **142a** and adipic acid of **142b** did not possess favorable properties as LAT1 substrates as **142c**, showing that translocation of the LAT1 prodrugs across the BBB is dependent on their affinity to LAT1. In addition to the in situ studies, **142c** was also taken up into the rat brain after its intravenous administration in vivo in rats. Peura and co-workers could not find enhanced concentrations of **141** in the rat striata after IV administration of **142c** when compared with the corresponding equimolar dose of **138**. Thus, authors hypothesized that **142c** is too quickly eliminated from the brain due to its high polarity [188]. As seen before with **119** [161], the attachment of phenylalanine to parent drug via an amide bond from meta-position of its aromatic ring could be a feasible promoiety for LAT1-targeting of cationic drugs [188].

#### 3.5.5. Neuropathic Pain

Glycine is the most abundant inhibitory neurotransmitter in the spinal cord [189]. Blockage of strychnine-sensitive glycine receptors, with strychnine, in the spinal cord elicits allodynia, a condition in which normally innocuous tactile stimuli provoke intensive aversive responses suggestive of pain [190]. Furthermore, blockage of glycine receptors by strychnine enhances neuropathic pain [191]. Intracerebroventricular intrathecal injection of glycine produces analgesia in thermal and chemical nociception [192] suggesting that glycine modulates pain responses to a variety of nociceptive *stimuli*.

**Figure 60:**
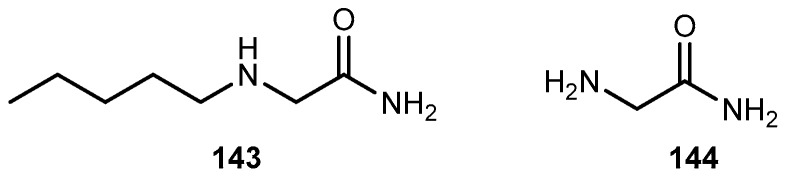
Chemical structures of melacemide (**143**) and glycinamide (**144**).

The potential therapeutic use of glycine as an analgesic has been hindered by its limited penetrability into CNS [193], as a large amount of the amino acid is being required to produce significant levels in CNS [194]. Some percursors like melacemide (2-*n*-pentyaminoacetamide) (**143**, Figure 60), readily penetrate into the CNS and elevate glycine concentrations in the brain [195]. This precursor is used as an antiepileptic drug and interferes with the production of allodynia by strychnine, and is metabolized primarily by monoamine oxidase (MAO-B) to glycinamide (**144**, Figure 60), which is the immediate precursor of glycine. Beyer and co-workers suggested that **144** is more effective than **143** in producing analgesia due to the magnitude duration, and effectiveness of the oral administrations rout, of this anti-nociceptive effect of **144** [196].

**Figure 61:**
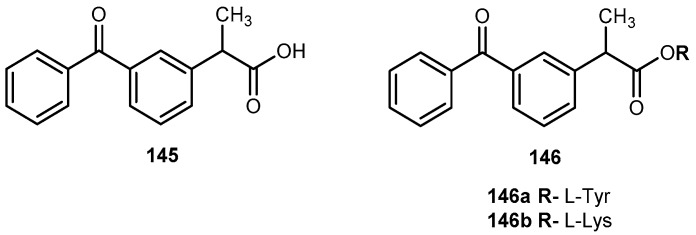
Chemical structures of ketoprofen (**145**) and its amino acid prodrugs.

Gynther and co-workers described a feasible means to achieve carrier-mediated drug transport into rat brain via LAT1, by conjugating a model compound to *L*-tyrosine and *L*-lysine (Figure 61). A hydrophilic drug, ketoprofen (**145**, Figure 61), that is not a substrate for LAT1, was chosen as a model compound [197]. This drug is used for the treatment of some nerve pain such as sciatica and postherpetic neuralgia.

The **146a** and **146b** prodrugs of **145** showed a concentration-dependent, yet saturable, brain uptake during in situ rat perfusion experiments. The uptake was inhibited by 2-aminobicyclo (2,2,1)heptan-2-carboxylic acid (BCH), a well-known LAT1 inhibitor, suggesting that the brain uptake of prodrugs was mediated by LAT1, even though **145** is not a LAT1 substrate. Probably the conjugation of amino acid and **145** turns prodrugs more suitable to link to LAT1. Furthermore, after bolus injection of **146b** in rats, both prodrugs and parent drug were detected in the whole rat brain tissue. The in vivo microdialysis in rat stratia studies showed that **146b** was able to deliver parent drug to its site of action, i.e., into intracellular compartments of brain cells [151,197].

#### 3.5.6. Depression

**Figure 62:**
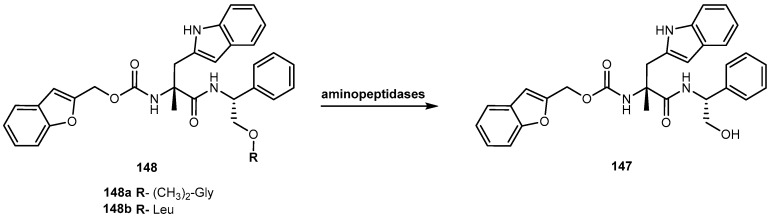
Bioconversion of amino acid ester prodrugs (**148a**) and CAM-4580 (**148b**) to its parent drug.

CAM-4451 (**147**, Figure 62) is a selective high-affinity NK_1_, neurokinin receptor, antagonist that is used as an antidepressant agent [97]. The **147** has a very low aqueous solubility (<2 μg/mL) and consequently poor oral bioavailability [97]. Chan and co-workers used a targeted prodrug strategy to increase the absorption of the poorly water-soluble parent drug, synthesizing amino acid ester prodrugs of **147** (**148a** and **b**, Figure 62). Bioconversion of prodrugs CAM-4562 (**148a**) and CAM-4580 (**148b**) by aminopeptidases to parent drug is depicted in Figure 62.

Amino acid leucine, **148b**, has a modest increase in solubility of **147**, while the dimethylglycine prodrug, **148a** has a 1500-fold greater solubility and nearly 3-times better oral bioavailability than of its parent drug [97]. Moreover, **148a** showed more selective hydrolysis compared to the parent drug by aminopeptidases at the brusher border membrane of the GI tract, whereas **148b** was more susceptible to hydrolysis before reaching the brush border membrane, which may cause potential precipitation problems after absorptions [97].

#### 3.5.7. Anaesthetic

**Figure 63:**
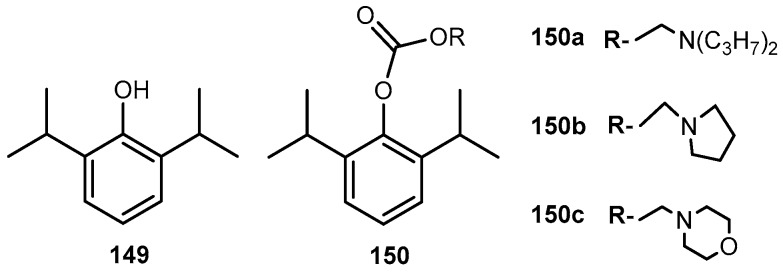
Chemical structure of amino acid derivatives of **149** [199].

Propofol (2,6-disopropylphenol) (**149**, Figure 63) is an intravenous agent used for anesthesia. Due to its very low solubility, **149** is formulated as an oil-water emulsion (1% *w*/*v*) of soya bean oil, glycerol and purified egg phosphatide (Diprivan^®^). As a lipid-based emulsion, it suffers from a number of limitations, such as poor physical stability, a potential for embolism, and need for strictly aseptic handling [198]. These drawbacks have stimulated an active research for safe alternative dosage forms, and, in particular, aqueous formulations.

Several α-amino acid ester derivatives (**150a**–**c**, Figure 63) were investigated by Trapani and co-workers. Generally, the derivatives showed low-to-moderate solubility and high stability in water solution [199].

The amino acid derivatives exhibited resistance in plasma and brain homogenates that were too high for them to consider them as true prodrugs. Interestingly, some of them have been found to interact with subtype A of GABA_A_ receptor, a major target mediating pharmacological actions of **149** and other general anesthetics [200].

**Figure 64:**
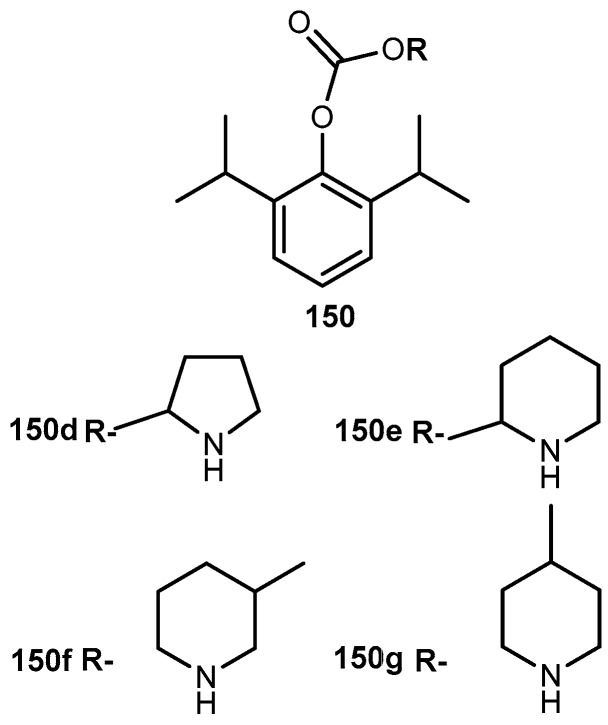
Cyclic amino acid derivates of propofol synthesized by Altomare et al. [201].

Different derivatives of **149** (**150d**–**g**, Figure 64) were prepared by coupling, via an ester bond, the drug with cyclic amino acids, namely *L*-proline (106 band positional isomers of piperidine carboxylic acid). Their solubility and stability in aqueous system solution, lipophilicity, susceptibility to enzymatic hydrolysis in animal plasma and liver, as well as their ability to modulate GABA_A_ receptors were evaluated [201].

The *L*-proline (**150d**) and racemic nipecotic acid (**150f**) esters were found to be highly soluble in water and sufficiently stable at physiological pH (half-lives > 6 h) whereas the α-amino acid esters (**150d** and **150e**) were found to be quantitatively hydrolyzed in plasma and liver esterase solutions, within a few minutes, showing prodrug behavior. The in vitro activity of esters demonstrated a mechanism involving allosteric modulation of GABA_A_ receptors. The in vivo anticonvulsant and anesthetic activities of prolinate **150d**, administered intraperitoneally in aqueous solution, made Altomare and co-workers consider this prodrug as a candidate for developing formulations useful for parenteral administration [201].

**Figure 65:**
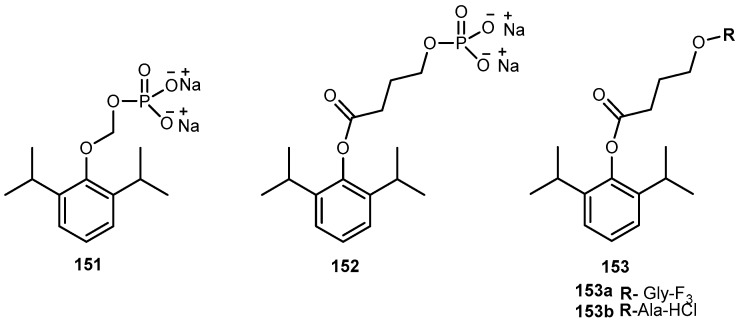
Chemical structures of phosphate and amino acids prodrugs of **149.**

Phosphate ester prodrugs of **149**, fosfopropofol (**151**) and HX0969w (**152**) (Figure 65) were designed to avoid the unsatisfactory water solubility of the parent drug. However, in previous clinical trials, there were reported prodrug side effects such as paresthesia and pruturius which might be related to the accumulation of phosphate ester [202]. To exclude this potential risk, Lang and co-workers designed two amino acid prodrugs, HX0969-Gly-F_3_ (**153a**) and HX0969-Ala-HCl (**153b**) (Figure 65), based on the lead compound **152**, by introducing the amino acid group into the structures of the prodrugs of **149** [203].

The hydrolysis assays in both rat and rhesus monkey plasma revealed that two amino acid prodrugs release parent drug to a greater extent at a more rapid rate than the phosphate prodrug during a period of five hours. All prodrugs released **149** rapidly in the presence of rat hepatic enzymes. The in vivo studies showed that amino acid prodrugs, administered intravenously could have a more rapid onset of action in a smaller dose than **151** and **152** [203]. Therefore, the application of the amino acid group to the prodrug of **149** could be a good strategy to improve water solubility and the onset of action.

## 4. Conclusions

In conclusion, amino acid prodrugs have a proven track of improving several pharmacological parameters, such as oral bioavailability of drugs that have poor solubility and permeability. More recently, amino acid prodrugs have successfully been used to achieve sustained release, intravenous delivery, and improved metabolic stability. Moreover, amino acid promoieties have been described as an important tool to enhance the transposition of the blood-barrier brain, which is important for several central nervous diseases that have few drugs available to combat them. In addition, these promoieties are safe pharmacological agents and do not increase the toxicity of compounds but might alleviate side effects of most drugs, especially those used as anticancer agents. We hope that this review stimulates the research on use of amino acid prodrug to overcome delivery changes and to discover effective new or improve older drugs.
